# Photo‐Driven CO_2_ Reduction With a Heterogenized Re Catalyst in the Metal–Organic Framework PCN‐777: Effects of Catalyst Loading and Anchoring Strategy on Catalysis

**DOI:** 10.1002/cssc.202502216

**Published:** 2026-01-27

**Authors:** Wojciech G. Sikorski, Martijn J. Mekkering, Arno van der Weijden, Stefania Tanase, Joost N. H. Reek, Jarl Ivar van der Vlugt

**Affiliations:** ^1^ Homogeneous, Supramolecular and Bioinspired Catalysis Group van ‘t Hoff Institute for Molecular Sciences Faculty of Science University of Amsterdam Amsterdam The Netherlands; ^2^ Heterogeneous Catalysis and Sustainable Chemistry Group van ‘t Hoff Institute for Molecular Sciences Faculty of Science University of Amsterdam Amsterdam The Netherlands; ^3^ Self Organizing Matter Group AMOLF Amsterdam Amsterdam The Netherlands; ^4^ Functional Materials Group van ‘t Hoff Institute for Molecular Sciences Faculty of Science University of Amsterdam Amsterdam The Netherlands; ^5^ Bioinspired Coordination Chemistry and Homogeneous Catalysis Group Institute of Chemistry School of Mathematics and Science Carl von Ossietzky University Oldenburg Oldenburg Germany

**Keywords:** CO_2_ photoreduction, functionalized metal–organic framework, heterogenized molecular catalyst

## Abstract

This study explores the impact of (i) different installation modes of the molecular rhenium catalyst **Re** within the PCN‐777 metal–organic framework (MOF) and (ii) catalyst loading on the resulting catalytic performance and recyclability of the different hybrid materials in the photocatalytic reduction of CO_2_ to CO. Through systematic investigation, we demonstrate that a robust coordination linkage between a zirconium node of the framework and the catalyst, obtained via solvent‐assisted ligand incorporation (SALI), minimizes rhenium leaching. In contrast, physical entrapment and electrostatic anchoring methods result in significant rhenium leaching after installation. Additionally, we reveal how the installation mode influences the electronic properties of the catalyst, which allows us to tune the catalytic activity. Furthermore, based on these results, we determine the optimal loading and concentration of **Re** within the MOF matrix for photocatalytic CO_2_ reduction.

## Graphical abstract

This study explores the impact of different installation modes of the molecular rhenium catalyst Re within the PCN‐777 metal–organic framework, comparing physisorption, electrostatic bindind and solvent‐assisted ligand incorporation (SALI), with the latter being the preferred installation mode in light of both catalytic performance and recyclability of the resulting hybrid materials for the photocatalytic reduction of CO_2_ to CO. Furthermore, the optimal Re loading and concentration within the MOF matrix is established using the SALI method.

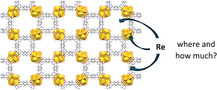

## 1 Introduction

The photocatalytic reduction of CO_2_ into more valuable and functional building blocks for chemical transformations is regarded as one of the most crucial areas of current catalytic research, essential for the transition toward a circular, sustainability‐based society [1, 2, 3]. Carbon monoxide has been identified as one of the attractive targets in this regard, as it can be fed into many different existing processes (drop‐in chemical) while also being an important starting material for many industrial‐scale processes. Molecular catalysts offer a precisely tailored coordination sphere, exhibit excellent selectivity [3, 4], and allow for fundamental insight and mechanistic understanding [5, 6, 7]. However, stability and recyclability of homogeneous catalysts limit their application on a larger scale [8, 9, 10, 11]. Hence, further efforts in material design and preparation are necessary to realize CO_2_ to CO conversion at the desired‐scale. Moreover, translating these molecular systems into “heterogenized” systems is deemed an essential next step [12].

Heterogenization of a well‐defined molecular catalyst by immobilizing it within a porous matrix of a metal–organic framework (MOF) constitutes a very promising approach to obtain a catalyst@MOF hybrid material that can combine the advantages of homogeneous (selectivity, activity, tunability) and heterogeneous (durability, recyclability) catalysis [12, 13, 14]. Installing a catalyst inherently renders the MOF scaffold less porous, hence hindering the diffusion of the reactants, although high catalyst loading ensures material with densely packed catalytic sites, which in turn prevents the usage of a vast amount of material. MOFs with macropores (pore size > 50 nm) would seem to be a logical and obvious solution to address this challenge [15, 16, 17]. However, these MOFs tend to collapse after solvent removal from their pores [18, 19, 20, 21]. An alternative approach is to design a framework with an intrinsic hierarchical architecture, i.e., a MOF featuring both micropores (pore size < 2 nm) and mesopores (2 nm < pore size < 50 nm), to separate the catalyst embedding within material from mass transport through the matrix [22, 23, 24, 25, 26, 27].

Furthermore, optimization of catalyst positioning and loading within a MOF may play a pivotal role in maximizing the performance of catalytically active materials [28]. It was reported that the catalytic site density can steer the selectivity of product formation in photocatalytic CO_2_ reduction [29]. Regarding the catalyst positioning, several strategies for catalyst heterogenization in MOFs exist, including physisorption, anchoring via electrostatic interactions and postsynthetic modification (PSM) methods [30, 31, 32, 33, 34, 35, 36, 37, 38, 39, 40, 41]. Solvent‐assisted ligand incorporation (SALI) is a specific PSM method that offers a very convenient and robust guest assembly through node grafting, and hence allows for a precise guest placement [42, 43, 44]. Common for all strategies to introduce an intact molecular species into a preassembled MOF structure is that the pore (window) size should be adequately large to allow unhindered diffusion of the (catalyst) species through the entire matrix. Each of these incorporation methods requires a different synthetic procedure to obtain the final hybrid material. Moreover, the spatial positioning (as well as anchoring connectivity) of the molecular entity within the porous MOF structure is not uniform between the methods. As a result, the guest molecule may be present in distinctly different parts of either the MOF cavity or near the nodes of the MOF framework and have relative rotational freedom or be fixed in place. This in turn, provides specific and potentially very different chemical and electronic microenvironments as well as confinement effects. These could affect subsequent interactions between catalyst and substrate and thereby influence catalyst performance.

To date, there are no studies that directly compare different installation modes of molecular catalysts into MOFs. Hence, potential differences in binding interactions of molecular catalysts within MOFs and their potential impact on catalysis remain unexplored. Such a comparative investigation could further contribute to the rational design of hybrid MOF‐molecular systems for the photocatalytic production of renewable fuels. We herein demonstrate, using PCN‐777 as an hierarchical metal‐organic framework material and the well‐defined rhenium complex (Re(4,4′‐bpydc)(CO)_3_Cl (**Re**) as a known catalyst for CO_2_ photoreduction [45, 46, 47, 48] (4,4′‐bpydc = 2,2′‐dipyridyl‐4,4′‐dicarboxylic acid), how different installation modes influence the porosity of the resulting MOF as well as the electronic properties of the catalyst itself. We further show to what extent the installation mode affects the performance of the anchored **Re**‐catalyst for the photocatalytic reduction of CO_2_ to CO. The SALI method yields the most active hybrid catalyst system. With the optimal anchoring strategy selected, we investigate the influence of catalyst loading and concentration of the **Re** catalyst anchored in PCN‐777 on the photocatalytic performance. It is found that a 1.8 wt% Re‐loading provides the highest activity for the photocatalytic reduction of CO_2_ to CO.

## 2 Results and Discussion

### 2.1 Selection of the Metal–Organic Framework Material

**FIGURE 1:**
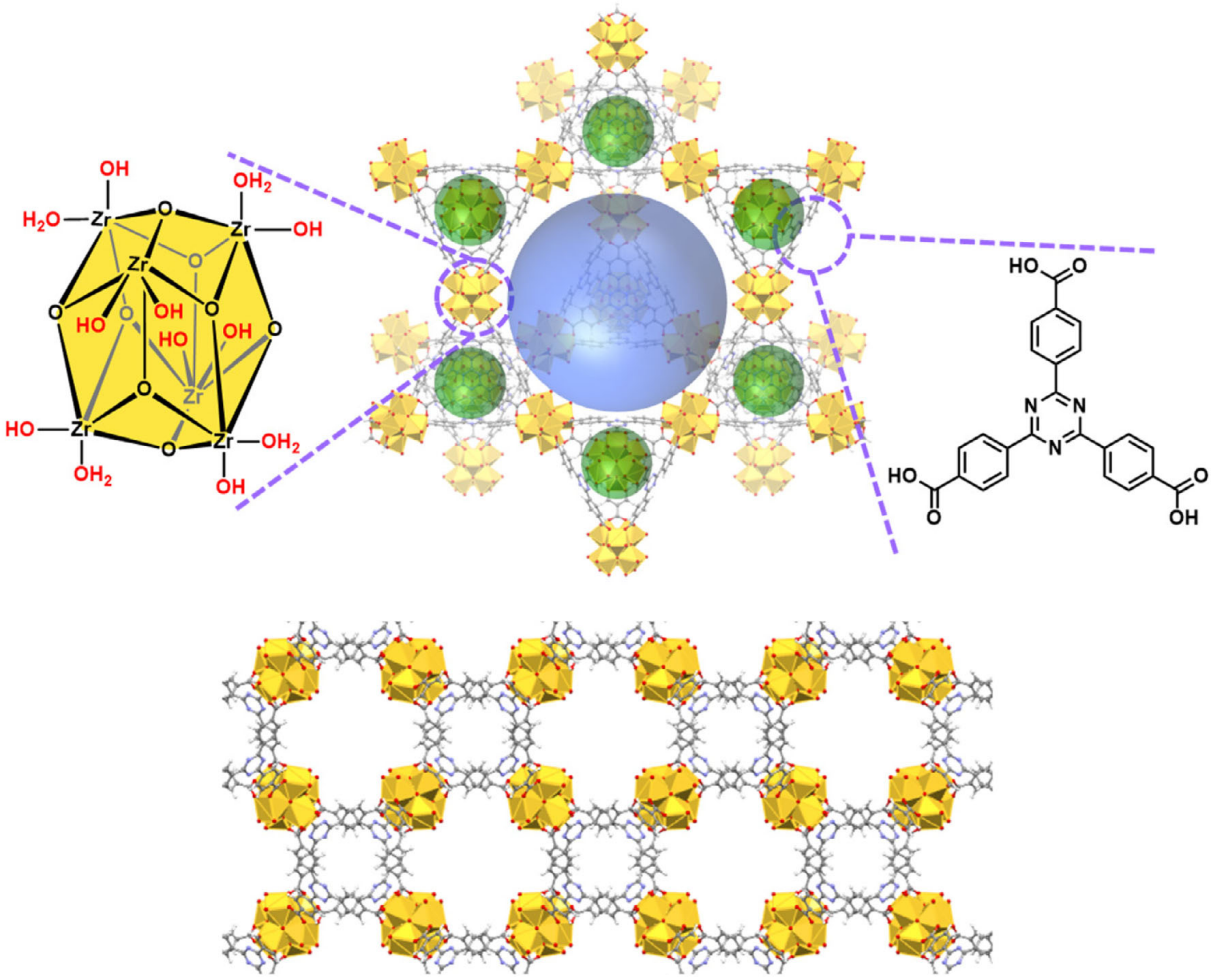
Top: Structure of the MOF PCN‐777 showing its Zr_6_ node and triazine‐based linker. The blue and green spheres represent different pore sizes (blue for the larger pores—38 Å; window size 30 × 35 Å, green for the smaller pores—9 Å; window size 8 × 14 Å). Bottom: The x‐axis view of PCN‐777, highlighting the framework's hierarchical porosity.

MOF PCN‐777 (Figure 1) is available via solvothermal synthesis [43, 49]. It consists of easily functionalized building blocks, shows high thermal and chemical stability and contains secondary building units (SBUs) that allow to chemically graft a catalyst [42]. Owing to its inherent mesoporous nature and functionalization capabilities, PCN‐777 has been investigated for adsorption and gas capture [50], sensing [51], chemical warfare and toxins degradation/removal [52, 53, 54], as well as ionic [55] and proton [56] conductivity. Applications in catalysis are scarce to date [57, 58, 59], especially when compared to other well‐studied zirconium MOFs (e.g., UiO series, NU‐1000, or porphyrinic PCN‐222/MOF‐545). This scarcity illustrates the opportunity for further exploration and development in this area.

Recent studies showed that immobilization of **Re** in several MOF materials (MIL‐101‐NH_2_(Al), UiO), could lead to suppressed unwanted degradation and deactivation pathways of the **Re** system, thereby enabling enhanced photocatalytic activity and stability in CO_2_ reduction [60, 61, 62]. We recently disclosed the use of a hybrid **Re**‐in‐PCN‐777 material in a dye‐sensitized photoelectrochemical CO_2_ reduction, using a novel heteroleptic cobaltocene derivative as redox mediator [63]. Nonetheless, to the best of our knowledge, a direct correlation of different anchoring strategies using the same MOF architecture, catalyst loading, and photocatalytic competence is missing. A very recent report examined the influence of different interactions between MOF and the rhenium catalyst (electrostatic vs*.* coordination) on CO_2_ photoreduction. It demonstrates that the microenvironment within the MOF can modulate the CO_2_ activation pathway [64]. However, the MOFs employed in these studies exhibit distinct topologies and are constructed from different building blocks, which could further influence the performance of the catalyst‐MOF systems.

### 2.2 Materials Characterization – Side‐by‐side Comparison of Anchoring Strategies

**FIGURE 2:**
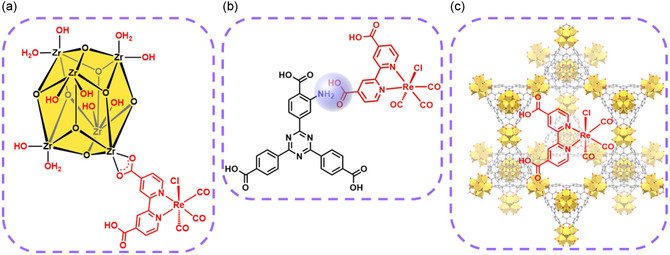
Conceptual representation of the rhenium catalyst installation modes within PCN‐777, (a) SALI Re@PCN‐777‐SALI, (b) electrostatic interaction Re@PCN‐777‐NH2, and (c) encapsulation via physisorption Re@PCN‐777.

**FIGURE 3:**
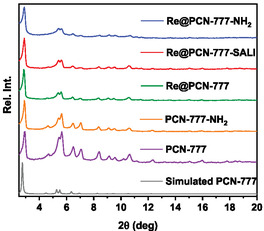
pXRD patterns of the as‐synthesized pristine PCN‐777 (with simulated spectrum), the PCN‐777‐NH2 precursor and the three **Re**‐in‐PCN‐777 hybrids.

The rhenium catalyst **Re** was introduced within the porous PCN‐777 host matrix via three different installation modes: (a) SALI (**Re**@PCN‐777‐SALI), (b) H‐bonding/electrostatic interaction, using an amine group‐functionalized triazine derivative to interact with one of the carboxylate groups in the backbone of the bipyridine ligand bound to the rhenium center (**Re**@PCN‐777‐NH_2_), and (c) physical encapsulation (**Re**@PCN‐777) (Figure 2). The **Re**@PCN‐777‐NH_2_ and **Re**@PCN‐777 materials were readily obtained from room temperature protocols, but catalyst installation via the SALI method required elevated temperature to facilitate exchange of the labile OH/H_2_O ligands by the ‐COOH functionality present in the bipyridyl ligand of the catalyst (see SI for details). Well‐defined signals were observed by powder X‐ray diffraction (pXRD) (Figure 3), which all overlap with the simulated PCN‐777 pattern, indicating retention of the MOF structure and crystallinity after the molecular rhenium complex was introduced, regardless of the method chosen. To the best of our knowledge, this is the first example of successful SALI carried out with the PCN‐777 metal organic framework.

**FIGURE 4:**
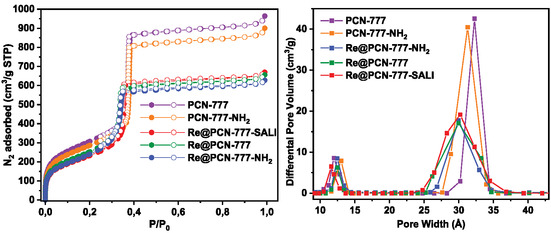
Left: N_2_ adsorption isotherms for PNC‐777, PCN‐777‐NH_2_, and **Re**‐in‐PCN‐777 hybrids. Right: Pore size distribution for PCN‐777, PCN‐777‐NH_2_, and Re‐in‐PCN‐777 hybrids.

Brunauer–Emmett–Teller (BET) analysis was performed using N_2_ adsorption isotherms to determine the respective surface areas (BET analysis for all materials was executed according to the two consistency criteria proposed by Rouquerol [65, 66]; Figures S15–S20). The adsorption isotherms exhibited a significant reduction in N_2_ uptake for each material, which implies successful incorporation of rhenium catalyst within the framework. Importantly, N_2_ uptake curves are very similar for all **Re**‐in‐PCN‐777 hybrid materials (Figure 4, left), suggesting that neither the type of interaction between the host and the guest, nor the potentially different location of **Re** within the MOF skeleton, significantly influences the material's porosity or external surface. This property is particularly crucial for the catalytic comparison studies, as markedly different porosities could heavily affect mass transport through the MOF and external surface area differences affect the active site density [67, 68]. The reduction in nitrogen adsorption primarily occurs at *P*/*P*_0_ = 0.4, corresponding to a mesoporous cage [35]. Moreover, pore size distribution analysis showed a proportional decrease of the pore volume with a slight contraction of the pore width (Figure 4, right) for the mesopores compared to the parent MOFs, but only a small change was observed for the smaller micropores present within the PCN‐777 matrix.

Based on these observations, it can be concluded that **Re** is situated within the larger pore for all three **Re**‐in‐PCN‐777 hybrids. The rhenium content (determined by inductively coupled plasma mass spectrometry (ICP‐MS)) in the thus obtained materials differed negligibly between the different installation modes (Table S1), which proves that nearly all **Re** molecules from the solution were incorporated within the MOF during the impregnation process, irrespective of the entrapment procedure.

**FIGURE 5:**
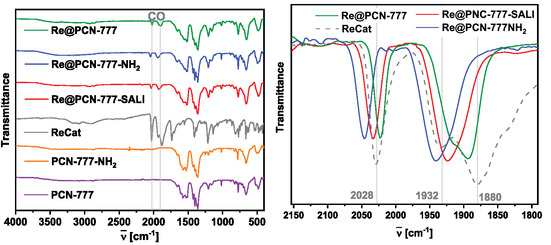
(Left) ATR‐FTIR spectra of pristine PCN‐777 and PCN‐777‐NH_2_, molecular **Re** and **Re**‐in‐PCN‐777 hybrids; (Right) Zoom‐in, CO region of **Re**‐in‐PCN‐777 hybrids and **Re**.

ATR‐IR spectroscopy (Figure 5) revealed two characteristic bands between 2050 and 1900 cm^−1^, which are attributed to CO stretching frequencies of complex **Re** ‐ pristine PCN‐777 showed no CO bands in this region. Different wavenumbers for the respective CO bands were observed for each material (see also Table S2 and Figure S14), with the physisorbed material showcasing the highest and **Re**@PCN‐777‐NH_2_ the lowest relative electron density at the Re‐center (i.e. the lowest and highest CO stretching frequencies, respectively), suggesting that the assembling mode of the rhenium complex within the MOF matrix influences the corresponding electronic properties of the Re‐center.

**FIGURE 6:**
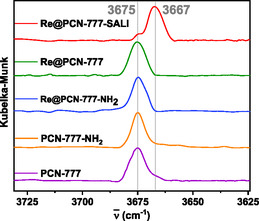
DRIFT spectra (‐OH region) for PCN‐777, PCN‐777‐NH_2_, and **Re**‐in‐PCN‐777 hybrids.

Diffuse reflectance infrared fourier transform (DRIFT)spectroscopy revealed subtle differences between **Re**@PCN‐777‐SALI and other hybrid materials regarding bands attributed to the ‐OH groups of the Zr cluster (Figures 6 and S26). The band at 3675 cm^−1^, attributed to terminal OH groups, remained unchanged after catalyst insertion within the framework for **Re**@PCN‐777‐NH_2_ and **Re**@PNC‐777. In contrast, the same signal underwent significant attenuation in **Re**@PCN‐777‐SALI while a new band appeared at 3667 cm^−1^, associated with bridging OH groups, in agreement with reports on SALI with the zirconium cluster in MOF NU‐1000 [20, 69]. As SALI leads to selective exchange of a terminal Zr‐nodal ‐OH group for a carboxylate unit present in the bipyridine ligand of **Re—**no reaction occurs at bridging ‐OH groups—this strongly suggests that grafting onto the Zr cluster occurred only with **Re**@PCN‐777‐SALI, while the other two procedures left the Zr nodes intact.

### 2.3 Photocatalytic CO_2_ Reduction – Effect of Anchoring Strategy

The heterogenized **Re** systems were evaluated in the photocatalytic CO_2_ reduction, using tris(2,2′‐bipyridyl)ruthenium(II) hexafluorophosphate (**RuPS**) as photosensitizer and dimethylphenylbenzimidazoline (**BIH**) as sacrificial proton and electron donor in CO_2_‐saturated acetonitrile (8 mL) under 450 nm illumination. All reactions only produced CO (^1^H NMR spectroscopy ruled out formate production), indicating that the different anchoring strategies do not influence the chemoselectivity for the CO_2_ reduction. Moreover, in all cases, the heterogenized catalyst outperforms the molecular **Re** (Table 1), likely due to inhibition of undesired dimerization and suppressed photodegradation of **Re** [70, 71, 72]. For this reason, minimized leaching during reaction is desired. However, the amount of CO produced by the respective hybrid **Re**‐in‐PCN‐777 materials in the first cycle differs remarkably. **Re**@PCN‐777‐NH_2_ reached the highest turnover number (TON = 585), whereas the physisorbed **Re**@PCN‐777 catalyst exhibited significantly lower activity (TON = 390).

**TABLE 1:** Catalytic results for CO_2_ photoreduction with **Re**‐in‐PCN‐777 hybrids. Conditions: 8 mL MeCN saturated with CO_2_, **RuPS/Re** = 5, **BIH/RuPS** = 200, 450 nm LED, 10 h.

Entry	Material	Re@PCN‐777, mg	Re, μmol	CO, mL	TON
1	**Re**@PCN‐777‐NH_2_	5	0.481	5.49	589
2	**Re**@PCN‐777‐SALI	5	0.483	4.69	498
3	**Re**@PCN‐777	5	0.478	3.66	390
4	**Re**	—	0.483	3.49	372

These observations suggest that the installation mode of **Re** hybrid material affects the performance in photocatalytic CO_2_ reduction, potentially related to different electronic characteristics of the catalyst within each hybrid material, as suggested by the IR spectroscopic data. The relative order observed for the IR stretching frequencies of **Re**, which could correlate to the reduction potential required for CO_2_ reduction, aligns with the initial TON values.

**FIGURE 7:**
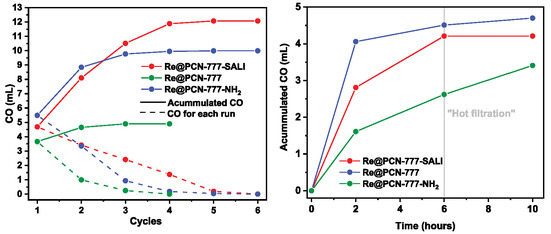
(Left) Iterative CO‐production (in mL) during each catalytic run (dashed line) and accumulated CO production (solid line) from recycling experiment. (Right) “redesigned” hot filtration test taken after 6 h of irradiation of the catalytic mixture (indicated by the gray line) with additional illumination of the collected supernatant for  4 h.

To comparatively evaluate the robustness of each anchoring methodology, recyclability experiments were performed (Figure 7, left). After the initial catalytic run, all solid material was isolated, washed, and then reused and recycled for five more catalytic runs, with fresh **BIH** and **RuPS** added after each recovery phase (During our investigations, we established that potentially entrapped **RuPS** photosensitizer did not to influence the catalytic performance of recycled material). **Re**@PCN‐777 could be used in only three catalytic cycles. This is in contrast to reactions carried out with **Re**@PCN‐777‐SALI and **Re**@PCN‐777‐NH_2_, where CO was detected over five catalytic runs (Figure 7, left, solid lines). Although the latter material showed almost no increase in cumulative CO production from cycles 4–6. Moreover, the activity loss varied for the different materials, with the most significant decline observed for **Re**@PCN‐777, followed by **Re**@PCN‐777‐NH_2_ and then **Re**@PCN‐777‐SALI. CO production decreased with each recycle for all three hybrids (dashed lines). The difference in the CO formation between the first and the second cycle equaled 2.67, 2.14, and 1.28 mL, respectively. Interestingly, **Re**@PCN‐777‐SALI outperformed **Re**@PCN‐777‐NH_2_ in CO production from the second catalytic run onward, with the difference in catalytic performance increasing over the next cycles. The total amount of CO formed by **Re**@PCN‐777‐SALI over six cycles (12.07 mL CO) is markedly higher than the productivity of **Re**@PCN‐777‐NH_2_ (9.98 mL CO) and **Re**@PCN‐777 (4.97 mL CO).

**FIGURE 8:**
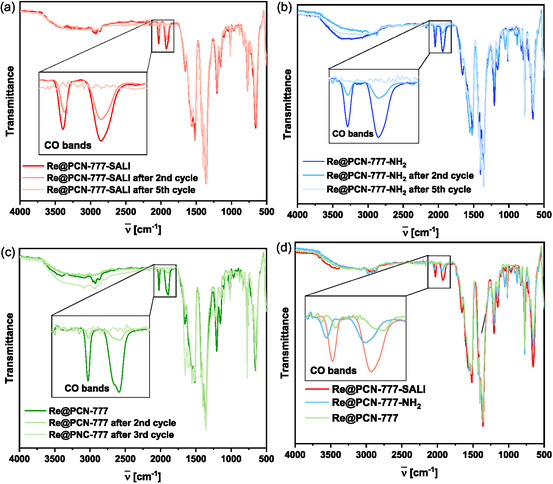
ATR‐FTIR spectra of (a) **Re**@PCN‐777‐SALI; (b), **Re**@PCN‐777‐NH_2_: (c) **Re**@PCN‐777 pre‐and postcatalysis (after 2^nd^ and 5^th^ cycle (3^rd^ for physisorbed material); (d) normalized comparison of IR spectra (inset: CO bands) of postcatalysis **Re**‐in‐PCN‐777 materials after 2^nd^ cycle.

A “hot filtration” test (Figure S28 – separation of the hybrid catalyst material by filtration from the reaction suspension after 2 h of irradiation, at which point the volume of CO formed was 4.06, 2.81, and 1.61 mL for **Re**@PCN‐777‐NH_2_, **Re**@PCN‐777‐SALI and **Re**@PNC‐777, respectively) showed no CO production by the mother liquor for the former two materials, but CO was detected for the entrapped catalyst material (0.33 mL). Hence, physisorption results in only partial heterogenization of **Re** in PCN‐777, with significant leaching of catalytically active material during catalysis. This makes physisorption the least efficient of the three installation modes. Infrared spectroscopy on the postcatalytic MOF materials obtained after the second cycle showed pronounced decreases in the intensity of the CO bands (of anchored **Re**) for both the electrostatically bound and physisorbed material, whereas the **Re**@PCN‐777‐SALI material exhibited relatively minor loss of signal (Figure 8). No CO stretching bands were observed in the IR spectra of any of the final materials recovered (i.e., after six cycles—three for the physisorbed material) (Figure 8a‐8c). However, the relative decrease in intensity was much more rapid for both **Re**@PCN‐777‐NH_2_ and **Re**@PCN‐777 compared to **Re**@PCN‐777‐SALI (Figure 8d). It is worth noting that the position of the CO bands in the postcatalytic solids varies between the different materials, similar to observations for the pristine hybrids.

Analysis of the solid materials recovered after the second and the last cycle by ICP‐MS (Table S3) showed that Re loading is stable for the **Re**@PCN‐777‐SALI samples but increasingly diminished rhenium content is observed for both **Re**@PCN‐777 and **Re**@PCN‐777‐NH_2_ samples, albeit at different rates. In light of these results, we reiterated the “hot filtration” test after a longer reaction time of 6 h, with the supernatant subjected to irradiation for an additional 4 h. Under these conditions, the mother liquor recovered from **Re**@PCN‐777‐NH_2_ proved competent for CO production (0.12 mL; Figure 7 right). This observation supports the leaching of **Re** species (in line with ICP analysis), albeit at a slower rate compared to **Re**@PCN‐777. Taken together, our comparative analysis of three installation modes illustrates different effectiveness and durability, with the SALI method outperforming both electrostatic interaction and physical entrapment. The observed loss in TON values over subsequent catalytic experiments likely stems from a chemical transformation at the Re‐center, attributed to the dissociation of CO ligands, as demonstrated by IR spectroscopy.

**FIGURE 9:**
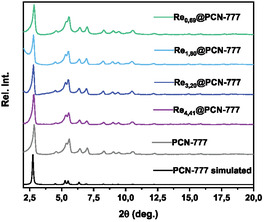
pXRD patterns of the as‐synthesized pristine PCN‐777, together with the simulated spectrum, and various Re_x_@PCN‐777 materials, where x denotes the wt% loading of Re.

### 2.4 Materials Characterization – Side‐by‐side Comparison of Catalyst Loadings

To assess whether the effect of catalyst loading on the photocatalytic reduction of CO_2_ with a heterogenized **Re**‐in‐PCN‐777 hybrid material, we prepared four **Re**_*x*_@PCN‐777 (x – Re weight%) derivatives *using the SALI method*. The rhenium content (x) in **Re**_*x*_@PCN‐777 materials was determined by ICP MS to range from 0.69 to 4.41 weight percent. This indicates that incorporation of the molecular Re‐catalyst proceeds with near‐quantitative efficiency in each case (Table S1). The crystalline structure of the as‐synthesized **Re**_*x*_@PCN‐777 materials and the retention of the PCN‐777 framework were confirmed by pXRD, showing well‐defined spectra that closely resemble the calculated pXRD pattern of pristine PCN‐777, irrespective of the rhenium loading (Figure 9). Scanning electron microscopy on the postsynthesis PCN‐777 materials did not show any morphological changes on the surface (Figures S9–S12). (EDX) Energy‐dispersive X‐ray spectroscopy elemental mapping did not offer direct evidence for a uniform distribution of the Re‐material throughout the sample, as there was a systematic decrease in reflections attributed to rhenium with lower catalyst loading.

**FIGURE 10:**
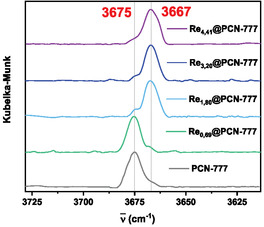
Zoom‐in of the range signifying terminal and bridging ‐OH groups in DRIFTS spectra of the parent PCN‐777 and **Re**_x_@PCN‐777 materials.

DRIFT spectroscopy showed a diminishing signal at 3675 cm^−1^, associated with terminal hydroxyl groups, as the rhenium content increased (i.e., with more ‐OH groups being substituted by carboxylate moieties). Simultaneously, the band at 3667 cm^−1^, associated with bridging hydroxyl groups, was uncovered (Figure 10). The material with the highest rhenium loading (**Re**_4.41_@PCN‐777) still contained free ‐OH groups, as the average loading is 0.7 rhenium catalyst per Zr6 node (calculation based on ICP‐MS, see Experimental Section).

Finally, BET analysis indicated that all materials remained hierarchical and mesoporous, with the characteristic S‐shaped IV‐type N_2_ isotherm indicating the presence of two different pore sizes (Figure S15) [73]. In agreement with our expectations, N_2_ uptake gradually went down with increasing catalyst content within the MOF pores. The material with the highest rhenium content (**Re**_4.41_@PCN‐777) can, however, still be qualified as (highly) porous, with a BET surface area that is ≈70% of the BET area measured for the pristine MOF. This porosity should facilitate mass transport of substrate and product during (photo) catalysis.

### 2.5 Photocatalytic CO_2_ Reduction – Effect of Catalyst Loading

The series of **Re**_*x*_@PCN‐777 hybrids were tested as catalysts for the photoreduction of CO_2_‐to‐CO, using the same conditions as applied for the initial study on the different catalyst installation modes (vide supra). The reactions with **Re**_*x*_@PCN‐777 were performed using 5 mg of material, irrespective of the Re content, in order to cancel out light scattering by the metal–organic framework. Hence, the rhenium catalyst “molarity” used for the catalysis, as well as the Re/Zr_6_ ratio, vary with different hybrid materials used. The hybrid material with 1.80 wt% rhenium (turnover number, TON = 501) outperformed the other systems by at least 100 TON, indicating that neither high nor low Re loading is beneficial for the reaction (Table 1, entries 1–4). The rationale behind the impact of excessive catalyst loading lies in its propensity to block pores [74], thereby hindering access to catalytic sites, especially those nestled deeper within the MOF framework, for substrates. Conversely, in materials with insufficient catalyst loading, photoexcited/reduced PS would, on average, need to traverse a longer path to reach the catalyst, potentially leading to energy dissipation. The homogeneous **Re** catalyst reached a lower TON per Re (TON = 372 – entry 5) than three out of the four investigated **Re**_*x*_@PCN‐777 hybrid materials. Similarly, the combination of pristine, nonfunctionalized PCN‐777 and the molecular **Re** catalyst dissolved in solution in the presence of **RuPS** and **BIH** resulted in a lower TON 347 (entry 6), as compared to the preassembled hybrids. In addition, **Re** immobilization strongly suppresses the formation of inactive rhenium dimer [60, 70] due to site isolation [75], hence extending the catalyst lifetime and suppressing undesired deactivation paths. This combination also performed slightly worse than the molecular catalyst alone, due to light scattering by the MOF material, diminishing the photoactivity. This set of experiments demonstrates that preinstallation is crucial for efficient CO_2_ photoreduction because this (i) leads to favorable confinement effects, resulting in longer‐lived catalytically active material and (ii) circumvents the need for diffusion of a molecular catalyst into the MOF matrix.

To further understand the correlation between the catalyst distribution and concentration within the MOF and the TON achieved, we compared the performance of three materials containing the same amount of **Re** as the 1.80 wt% sample (0.48 μmol) but differing in their intrinsic rhenium loadings (Table 2, entries 3 and 7–9). Very similar TONs were observed for the different materials, irrespective of the overall amount of catalytic hybrid material used (entries 1, 2, and 4), with slight differences attributed to varying degrees of light scattering consequences. Increasing the absolute amount of **Re** by using more of **Re**_1.80_@PCN‐777 (12.2 mg, 1.18 μmol, i.e., the same molar amount as present in 5 mg of **Re**_4.41_@PCN‐777 (entry 10)) gave the same high TON for this particular system. Increasing the amount of **Re**_4.41_@PCN‐777 used to 10.5 mg (i.e., 2.5 μmol of recatalyst) did not improve the TON for this catalyst material (entry 11). Taken together, these results show that (i) catalytic performance is not dependent on the catalyst concentration in the reaction mixture, and that (ii) catalytic performance is dependent on the catalyst loading within the PCN‐777 hybrid material. Using an iterative recycling strategy (5 cycles), the **Re**_1.80_@PCN‐777 material reached a total turnover number of 1289 after a cumulative period of 50 h (Figure S14).

**TABLE 2:** Catalytic results for CO_2_ photoreduction. Conditions: 8 mL MeCN saturated with CO_2_, Re_*x*_@PCN‐777 (amount in the table), RuPS/Re = 5, BIH/RuPS = 200, 450 nm LED, 10 h.

Entry	Material	Re_*x*_@PCN‐777, mg	Re amount, μmol	TON
1	**Re**_4.41_@PCN‐777	5	1.18	360
2	**Re**_3.20_@PCN‐777	5	0.86	399
3	**Re**_1.80_@PCN‐777	5	0.48	501
4	**Re**_0.59_@PCN‐777	5	0.16	226
5	**Re**	—	0.48	372
6	**Re** + PCN‐777	5 (PCN‐777)	0.48	347
7	**Re**_4.41_@PCN‐777	2	0.48	364
8	**Re**_3.20_@PCN‐777	2.8	0.48	403
9	**Re**_0.59_@PCN‐777	15.2	0.48	215
10	**Re**_1.80_@PCN‐777	12.2	1.18	493
11	**Re**_4.41_@PCN‐777	10.5	2.5	354

## 3 Conclusions

This study reveals valuable insights into the performance and stability of different installation modes of **Re** within PCN‐777 for the photocatalytic reduction of CO_2_ to CO. A coordination linkage between the Zr‐node and the catalyst ‐ as obtained by SALI ‐ enhances stability, while electrostatic interaction or physical entrapment leads to undesired rhenium leaching and decreased performance. This highlights the influence of the installation mode on the electronic properties of the catalyst within the MOF matrix, which potentially affects the catalytic performance. To the best of our knowledge, this is the first direct comparison of different installation modes of a catalyst within an MOF matrix in the literature.

Furthermore, we demonstrate that when the catalyst loading is maintained constant across all materials, the porosity remains unaffected by the different installation modes. The SALI method emerges as the most promising to ensure robust and durable guest assembly. We are not aware of any other example of successful SALI carried out with the PCN‐777 MOF.

Additionally, we find that while the anchoring mode does not affect the chemoselectivity observed for the CO_2_ photoreduction, it impacts both the catalytic production of CO and the durability of the anchored **Re** system. Rhenium catalyst loading influences the MOF porosity and strongly affects the MOF hybrid material performance in photocatalytic reduction of CO_2_ using an external Ru‐photosensitizer. The reaction is not dependent on an absolute rhenium concentration in the reaction mixture, but strongly depends on the catalyst loading within the functionalized hybrid MOF. The optimal loading of the molecular **Re** system, anchored via the Zr‐node using the SALI method, is 1.80 wt%. This material, **Re**_1.80_@PCN‐777, can be easily recovered and reused over five cycles. Overall, our findings emphasize the importance of thoughtful catalyst‐MOF integration for optimal performance in renewable energy applications.

## Supporting Information

Additional supporting information can be found online in the Supporting Information section. **Supporting** **Fig.** **S1:** ^1^H NMR spectrum of **Re(4,4’‐** **bpydc)(CO)_3_Cl** complex (Re) in DMSO‐d_6_. **Supporting Fig. S2:** ^13^C NMR spectrum of **Re(4,4’‐** **bpydc)(CO)_3_Cl** complex (Re) in DMSO‐d_6_. **Supporting Fig. S3:** Schematic synthetic procedure of H_3_TATB‐NH_2_ linker of PCN‐777‐NH_2_. **Supporting Fig. S4:** ^1^H NMR spectrum of **H_3_TATB‐NH_2_** in DMSO‐d_6_. **Supporting Fig. S5:** ^13^C NMR spectrum of **H_3_TATB‐NH_2_** in DMSO‐d_6_. **Supporting Fig. S6:** ^19^F NMR spectrum of digested PCN‐777 in D_2_SO_4_ + DMSO‐d_6_ before (blue) and after (red) the acid treatment. **Supporting Fig. S7:** UV‐Vis spectrum of a DMF solution of **Re** and consecutive supernatants from the immobilization process for **Re**_4,41_@PCN‐777. **Supporting Fig. S8:** Photographs of the catalyst immobilization mixtures and the final dry solid materials with different **Re** loading. **Supporting Fig. S9:** SEM images (top) and EDX elemental mapping for Zr and Re (bottom) in **Re**_4.41_@PCN‐777. **Supporting Fig. S10:** SEM images (top) and EDX elemental mapping for Zr and Re (bottom) in **Re**_3.20_@PCN‐777. **Supporting Fig. S11:** SEM images (top) and EDX elemental mapping for Zr and Re (bottom) in **Re**_1.80_@PCN‐777. **Supporting Fig. S12:** SEM images (top) and EDX elemental mapping for Zr and Re (bottom) in **Re**_0.69_@PCN‐777. **Supporting Fig. S13:** ATR‐FTIR spectra of pristine PCN‐777, the molecular rhenium catalyst **Re** and various **Re**x@PCN‐777 hybrids. **Supporting Fig. S14:** Zoom‐in of AIR‐IR spectra for CO bands of **Re**@PCN‐777 hybrids and **Re** (top). Schematic representation of the donating character of **Re** installation mode within PCN‐777. **Supporting Fig. S15:** Top: N2 adsorption isotherms for PNC‐777 and **Re**_x_@PCN‐777 hybrids (empty des. fill ads.). Bottom: Pore size distribution for PCN‐777 and **Re**_x_@PCN‐777 hybrids. **Supporting Fig. 16:** Porosity analysis of the pristine PCN‐777 a) Rouquerol transform plot, b) N_2_ adsorption isotherm, c) BET transform plot. **Supporting Fig. 17:** Porosity analysis of the pristine PCN‐777‐NH_2_ a) Rouquerol transform plot, b) N_2_ adsorption isotherm, c) BET transform plot. **Supporting Fig. S18:** Porosity analysis of **Re**@PCN‐777‐SALI a) Rouquerol transform plot, b) N_2_ adsorption isotherm, c) BET transform plot. **Supporting Fig. S19:** Porosity analysis of **Re**@PCN‐777‐NH2 a) Rouquerol transform plot, b) N_2_ adsorption isotherm, c) BET transform plot. **Supporting Fig. S20:** Porosity analysis of **Re**@PCN‐777 a) Rouquerol transform plot, b) N_2_ adsorption isotherm, c) BET transform plot. **Supporting Fig. S21:** Porosity analysis of **Re**_4.41_@PCN‐777 a) Rouquerol transform plot, b) N_2_ adsorption isotherm, c) BET transform plot. **Supporting Fig. S22:** Porosity analysis of **Re**_3.20_@PCN‐777 a) Rouquerol transform plot, b) N_2_ adsorption isotherm, c) BET transform plot. **Supporting Fig. S23:** Porosity analysis of **Re**_1.80_@PCN‐777 a) Rouquerol transform plot, b) N2 adsorption isotherm, c) BET transform plot. **Supporting Fig. S24:** Porosity analysis of **Re**_0.69_@PCN‐777 a) Rouquerol transform plot, b) N2 adsorption isotherm, c) BET transform plot. **Supporting Fig. S25:** BET area of **Re**_x_@PCN‐777 hybrids. **Supporting Fig. S26:** Full DRIFTS spectra for PCN‐777, PCN‐777‐NH2 and **Re**‐in‐PCN‐777 hybrids. **Supporting Fig. S27:** "UFO reactor" for photocatalytic reactions. **Supporting Fig. S28:** “Hot filtration” test taken after two hours of irradiation of the catalytic mixture (indicated by the grey line). **Supporting Fig. S29:** TON values for each run (dashed line) and accumulated TON (solid line) obtained with Re_1.80_@PCN‐777 in the recyclability experiment. Conditions: 8 mL MeCN saturated with CO_2_, **Re**_1.80_@PCN‐777 (recovered),RuPS/Re = 5, BIH/RuPS = 200, 450 nm LED, 10 h for each catalytic cycle. A new portion of the RuPS was added after every cycle. **Supporting Fig. S30:** ATR‐FTIR spectra of pre‐and post‐catalysis with **Re**_1.80_@PCN‐777. **Supporting Fig. S31:** ATR‐FTIR spectra of **Re**_1.80_@PCN‐777 after illumination (450 nm). **Supporting Fig. S32:** CO_2_ adsorption isotherms for PNC‐777 and Rex@PCN‐777 hybrids. **Supporting Fig. S33:** GC calibration curve for photocatalysis results. **Supporting Table S1:** Rhenium and zirconium content (weight percent wt%) in **Re**_x_@PCN‐777 hybrids from ICP‐MS results, number of the catalyst per Zr‐node of PNC‐777 and molar concentration of Re in DMF mother solution for catalyst impregnation. **Supporting Table S2:** CO peaks of various **Re**‐in‐PCN‐777 hybrids vs. the homogenous rhenium complex **Re**. **Supporting Table S3:** Rhenium and zirconium content (weight percent wt%) in Re‐in‐PCN‐777 hybrids before and after catalysis from ICP‐MS results.

## Conflicts of Interest

The authors declare no conflicts of interest.

## Supporting information

Supplementary Material

## Data Availability

The data supporting this article have been included in the main article and as part of the SI.
