# Supplementary material for: Photo‐Driven CO2 Reduction With a Heterogenized Re Catalyst in the Metal–Organic Framework PCN‐777: Effects of Catalyst Loading and Anchoring Strategy on Catalysis
Source: ChemSusChem. 2026 Jan 27;19(2):e202502216. doi: 10.1002/cssc.202502216 (PMC12840846; doi:10.1002/cssc.202502216)
Supplement: Supplementary file 1 — Supplementary Material [file CSSC-19-e202502216-s001.pdf]

**Supporting Information for**

# Photo-driven $CO_2$ reduction with a heterogenized Re catalyst in the metal-organic framework PCN-777 – Effects of catalyst loading and anchoring strategy on catalysis

Wojciech G. Sikorski, Martijn J. Mekkering, Arno van der Weijden, Stefania Tanase, Joost N. H. Reek, Jarl Ivar van der Vlugt*

## General and Methods:

**Chemicals:** All purchased chemicals were used without further purification.

**Powder X-ray diffraction (pXRD)** measurements (2-40$^{o}$, 2$^{o}$ min$^{-1}$) were performed on a silicon single-crystal wafer using Rigaku Miniflex X-ray Diffractometer, X-ray Ni-filtered Cu Kα radiation (λ = 1. 541874 Å). The X-ray tube was operated at 30 kV and 15 mA. The simulated powder X-ray diffraction (pXRD) patterns for PCN-777 (and its **Re**-functionalized derivatives) were obtained using the CIF file provided in the original publication[S1] and processed with Mercury software.

**Infrared (IR)** spectra were recorded on Bruker Alpha FTIR spectrometer.

**Diffuse reflectance infrared Fourier transform spectroscopy (DRIFTS)** was performed on a Nicolet iS50 FT-IT spectrometer (Thermo Fisher), equipped with a nitrogen cooled mercury-cadmium-telluride detector (MCT) and a KBr beamsplitter. Samples were diluted to approximately 5 wt% with dry KBr powder. A 4 cm$^{-1}$ resolution was used and the samples were scanned between 400–4000 cm$^{-1}$.

**Adsorption-desorption measurements** with $N_2$ at 77K and $CO_2$ at 298K were carried out on a Belsorp-MaxII analyser from *MicrotracBEL Corp.* Samples (around 50 mg) were pretreated under high vacuum and elevated temperature

in a Belprep-vacIII prior to analysis. The pre-treatment conditions were as follows: for PCN-777: 120 °C, 12 hours, for $Re_x$PCN-777: 65 °C, 16 hours. The specific surface area (SSA) was calculated based on the Brunauer-Emmett-Teller (BET) model using measured $N_2$ isotherms at 77K. BET analysis was performed according to the Rouquerol consistency criteria[S2] and all measured samples satisfied the Rouquerol criteria.

**Pore size distribution** (PSD) was derived from non-local density functional theory methods (NL-DFT) based methods using BELMaster 7 software.

**UV-Vis spectra** were recorded at room temperature using a Shimadzu UV-Vis spectrophotometer (UV-2600) in a 10 mm quartz cuvettes.

**Carbon monoxide quantification** was performed using a gas chromatograph (Shimadzu Nexis GC-2030) with a 5Å molecular sieves column (60 m, 0.32 mm internal diameter, 25 µm film thickness, at 40°C column temperature, a flow helium rate 5.5 mL/min), equipped with the thermal conductivity detector (TCD, current 75 mA, temperature 200°C).

**Inductively coupled plasma mass spectrometry** (ICP-MS) analysis was performed by Mikroanalytisches Labor Kolbe (Oberhausen, Germany).

**Scanning electron microscope** (SEM) and **Energy-dispersive X-ray spectroscopy** (EDX). The morphology of the materials was studied by using SEM (FEI Verios 460 scanning electron microscope) operated at 5 kV. The preparation of these materials involved the dispersion of polycrystalline material in acetonitrile, and the deposition of the resulting solution onto silicon substrates. EDX was measured using an Oxford X–Max$^N$ energy dispersive X-ray spectrometer with an accelerating voltage of 20 kV using a 50 pA current.

**Nuclear magnetic resonance (NMR)** spectra were recorded on a Bruker AMX 400 spectrometer at room temperature (chemical sifts are reported in ppm relative to $SiMe_4$ and the residual solvent signals.

Synthetic procedures: **Re(4,4'-bpydc)(CO)$_3$Cl (Re)** was obtained according the literature[S3] procedure with slight modifications. Re(CO)$_5$Cl (181 mg, 0.5 mmol) and 2,2'-dipyridyl-4,4'-dicarboxylic acid (122 mg, 0.5 mmol) were dissolved in the mixture of toluene (25 mL) and methanol (15 mL). The mixture was heated at reflux for 4 hours. Thereafter, the volume of solvent was reduced using a rotary evaporator. The precipitated, intensely orange colored solid was isolated by filtration and then washed with diethyl ether (3 × 15 mL). The remaining solid was dried under reduced pressure at room temperature. $^1$H NMR (400 MHz, DMSO-d$_6$): δ (ppm) = 14.40 (br. s, 2H), 9.21 (d, $^3J$ = 5,6 Hz, 2H), 9.14 (s, 2H), 8.13 (d, $^3J$ = 5,6 Hz, 2H); $^{13}$C NMR (400 MHz, DMSO-d$_6$): δ (ppm) = 197.45, 189.25, 164.25, 155.77, 154.06, 141.68, 127.08, 124.04

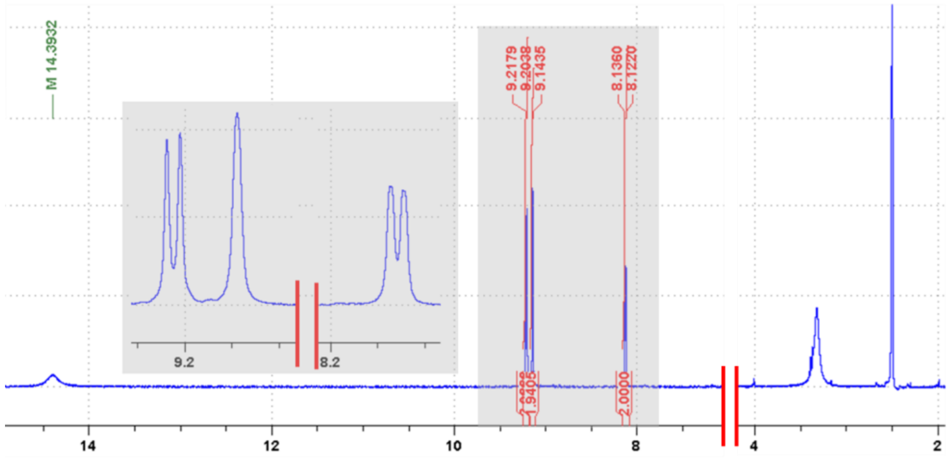


**Figure S1** $^1$H NMR spectrum of **Re(4,4'-bpydc)(CO)$_3$Cl** complex **(Re)** in DMSO-d$_6$

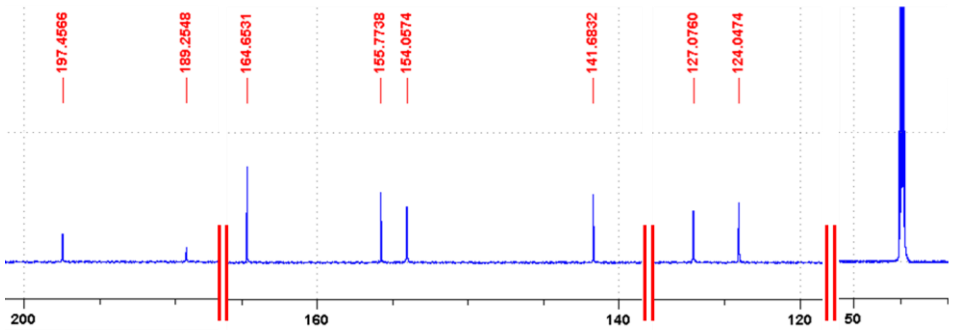


**Figure S2** $^{13}$C NMR spectrum of **Re(4,4'-bpydc)(CO)$_3$Cl** complex **(Re)** in DMSO-d$_6$

**2-(3-amino-4-carboxyphenyl)-4,6-bis(4-carboxyphenyl)-1,3,5-triazine** (**$H_3$TATB-$NH_2$**, linker for PCN-777-$NH_2$) was synthesized according to the literature[S4] with slight modifications.

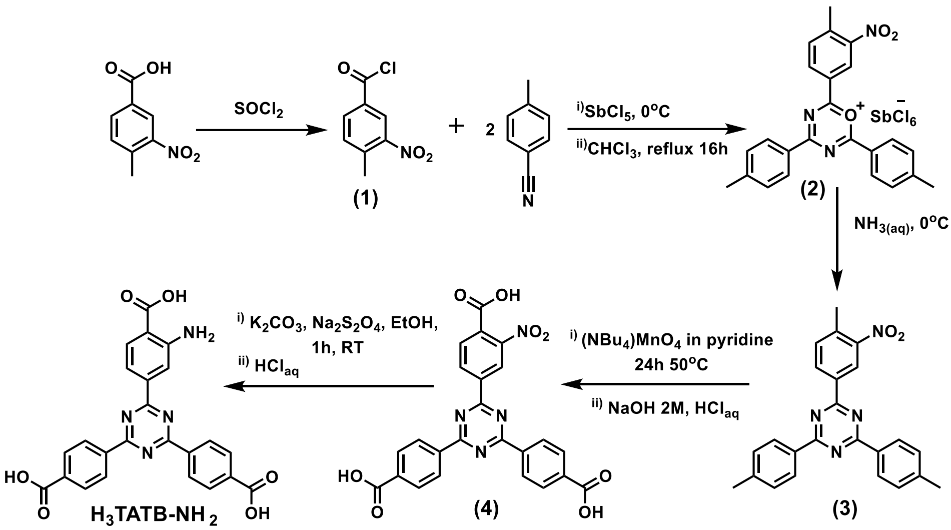


**Figure S3** Schematic synthetic procedure of $H_3$TATB-$NH_2$ linker of PCN-777-$NH_2$.

DMF (20 μL) was added to a suspension of 4-methyl-3-nitrobenzoic acid (3.00 g, 16.56 mmol) in thionyl chloride (15 mL). The mixture was heated to reflux for 2 hours, whereafter excess thionyl chloride was removed under reduced pressure to leave a yellow oil of 4-methyl-3-nitrobenzoyl chloride **1** that was used in the next step without further purification. To the mixture of crude **1** and 4-methylbenzonitrile (3.88 g, 33.12 mmol) in dry chloroform (35 mL) was added antimony (V) chloride (2.52 mL, 19.90 mmol) at $0^{o}$C under an $N_2$ flow. The mixture was heated to reflux for 16 hours and the formed yellow oxonium salt **2** was isolated by filtration. The obtained yellow solid was added in small portions to a solution of aqueous ammonia (28%, 200 mL) at $0^{o}$C and the mixture was stirred at room temperature for 2 hours. The obtained white solid was filtered off and then extracted with boiling chloroform (3 × 85 mL). The solvent was removed under reduced pressure to leave 2-(4-methyl-3-nitrophenyl)-4,6,-bis(4-methylphenyl)-1,2,3triazine **3** as a white solid in 83 % yield.

Freshly obtained tetrabutylammonium permanganate (5.52 g, 15.24 mmol) was added to a solution of **3** (1.25 g, 3.15 mmol) in pyridine (20 mL). The mixture was stirred at 50 °C for 24 hours before an aqueous solution of NaOH (2 M, 50 mL) was added to the brown slurry, resulting in the formation of a precipitate. The mixture was filtered and the filtrate was washed with *tert*-butyl methyl ether (3 × 75 mL) and finally treated with concentrated hydrochloric acid, stirred for 30 minutes and filtered to yield 2-(4-carboxy-2-nitrophenyl)-4,6-bis(4-carboxyphenyl)-1,3,5-triazine **4** as a yellowish solid (yield 73 %).

Solid **4** (1.1 g, 2.26 mmol) and potassium carbonate were suspended in water (40 mL) and stirred until everything dissolved. Ethanol (15 mL) was added to the solution and then sodium dithionite (4.62 g, 12.6 mmol, 10 equiv.). The mixture was stirred for 1 hour at room temperature. Ethanol was removed under reduced pressure and the residue was poured into concentrated hydrochloric acid at 0 °C. The precipitate was filtered, washed plentifully with water and dried in a vacuum oven to give **$H_3$TATB-$NH_2$** as an orange solid (78 %). **$^1$H NMR (400 MHz, DMSO-$d_6$):** δ (ppm) = 8.82 (d, $^3J$ = 8.5 Hz, 4H), 8.24 (d, $^4J$ = 1.2 Hz, 1H), 8.20 (d, $^3J$ = 8.5 Hz, 4H), 7.95 (d, $^3J$ = 8.4 Hz, 1H), 7.83 (dd, $^3J$ = 8.4 Hz, $^4J$ = 1.2 Hz, 1H). **$^{13}$C NMR (500 MHz, DMSO-$d_6$):** δ (ppm) = 171.0, 170.4, 169.2, 166.8, 151.5, 139.4, 138.9, 134.6, 131.8, 129.8, 128.8, 117.1, 114.4, 113.0.

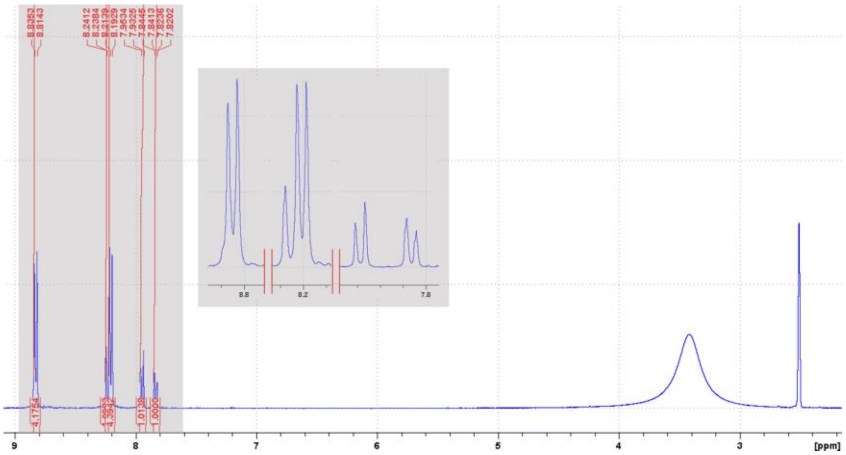


**Figure S4** $^{1}$H NMR spectrum **of $H_3$TATB-NH$_2$** in DMSO-$d_6$

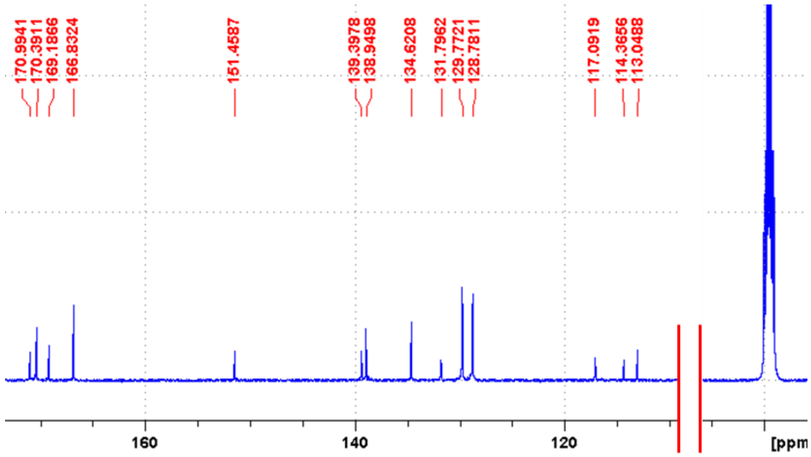


**Figure S5** $^{13}$C NMR spectrum of **$H_3$TATB-NH$_2$** in DMSO-$d_6$

**MOF PCN-777** was synthesized according to the literature procedure.[S1] However, the as-synthesized MOF contains node-blocking $CF_3COO^-$ ligands,[S5] as revealed by $^{19}F$ NMR analysis of the digested sample ($D_2SO_4$ + DMSO-$d_6$). To replace the acetate ligands with an $OH/H_2O$ pair (which can be substituted by **Re** in the SALI procedure), PCN-777 was ultrasonically suspended in HCl (0.5 M, 10 mL) and heated at 80°C for 12 hours. The powder was then washed with water and soaked in acetone three times, with the solvent exchanged every 12 hours. Following this acid activation, $^{19}F$ NMR of the digested MOF sample showed no trace of the acetate, indicating successful ligand replacement.

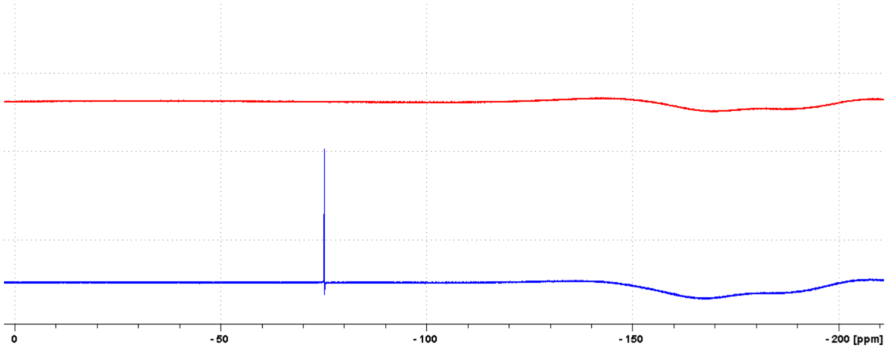


**Figure S6** $^{19}F$ NMR spectrum of digested PCN-777 in $D_2SO_4$ + DMSO-$d_6$ before (blue) and after (red) the acid treatment

**PCN-777-NH$_2$** was synthesized using an analogous procedure to PCN-777 employing **$H_3$TATB-NH$_2$** instead of $H_3$TATB. PCN-777-NH$_2$ was obtained as a pale yellow solid.

**Re@PCN-777-SALI**

PCN-777 (25 mg) was thermally activated at 120 $^{o}$C for 6 h. under reduced pressure and then added into a reaction vial (10 mL) containing the appropriate concentration of **Re** solution in dry DMF (5 mL) (Table S1). The vial was sonicated for 2 minutes and then placed into a preheated oven at 60 $^{o}$C. The supernatant was monitored with UV-Vis spectroscopy, specifically probing the absorption band at 390 nm, throughout the immobilization process. A rapid decay in absorption was observed after 1h, indicating an efficient diffusion of **Re** from solution into MOF pores, nevertheless the impregnation was continued for two more hours to ensure maximum MOF functionalization and to facilitate an even distribution of catalytically competent Re-species throughout the scaffold. After 3 hours, the vial was removed, the contents were allowed to cool down to room temperature and then centrifuged. The colourless supernatant was discarded and the solid was washed with fresh DMF (3 × 15 mL over 4 hours) and subsequently soaked in acetone (15 mL) for 24 hours, during which time the acetone medium was exchanged three times. The solid was dried at 50 $^{o}$C under reduced pressure.

**Re@PCN-777** and **Re@PCN-777-NH$_2$** were prepared in a similar procedure but the installation of **Re** was carried out at room temperature and in the case of **Re@PCN-777-NH$_2$**, PCN-777-NH$_2$ was used instead of PCN-777.

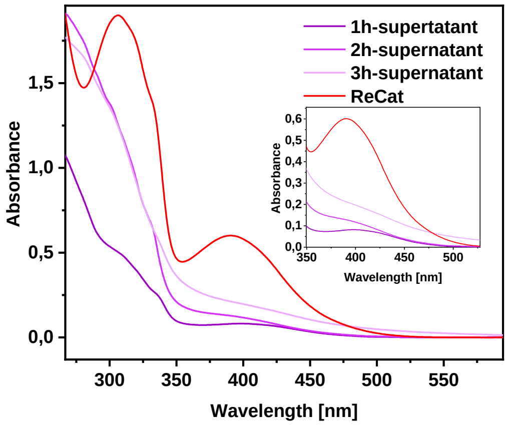


**Figure S7** UV-Vis spectrum of a DMF solution of **Re** and consecutive supernatants from the immobilization process for $\mathbf{Re}_{4,41}$@PCN-777

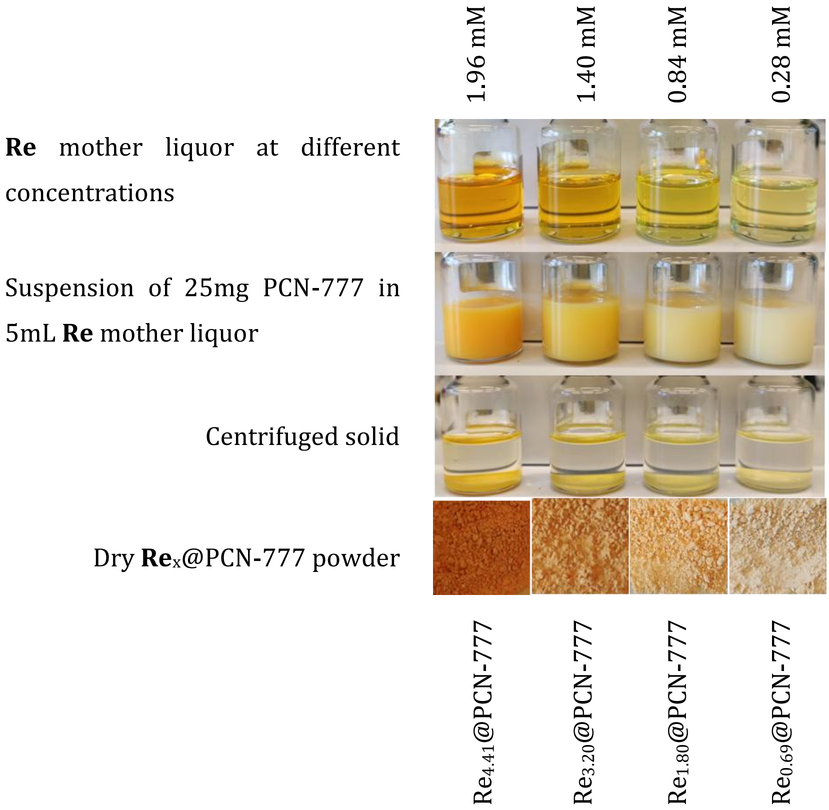


**Figure S8** Photographs of the catalyst immobilization mixtures and the final dry solid materials with different **Re** loading

**Table S1** Rhenium and zirconium content (weight percent wt%) in **Re$_x$**@PCN-777 hybrids from ICP-MS results, number of the catalyst per Zr-node of PNC-777 and molar concentration of Re in DMF mother solution for catalyst impregnation

| **Entry** | **Material** | **Re [mM]** | **ICP-MS** | | **Re/Zr$_6$** |
|---|---|---|---|---|---|
| | | | **Re [wt%]** | **Zr [wt%]** | |
| **1** | **Re$_{4,41}$**@PCN-777 | 1.96 | 4.41 | 18.35 | 0.70 |
| **2** | **Re$_{3,20}$**@PCN-777 | 1.40 | 3.20 | 19.82 | 0.47 |
| **3** | **Re$_{1,80}$**@PCN-777 | 0.84 | 1.80 | 18.25 | 0.29 |
| **4** | **Re$_{0,69}$**@PCN-777 | 0.28 | 0.69 | 21.37 | 0.095 |

**SEM images and EDX mapping for Re$_x$@PCN-777**

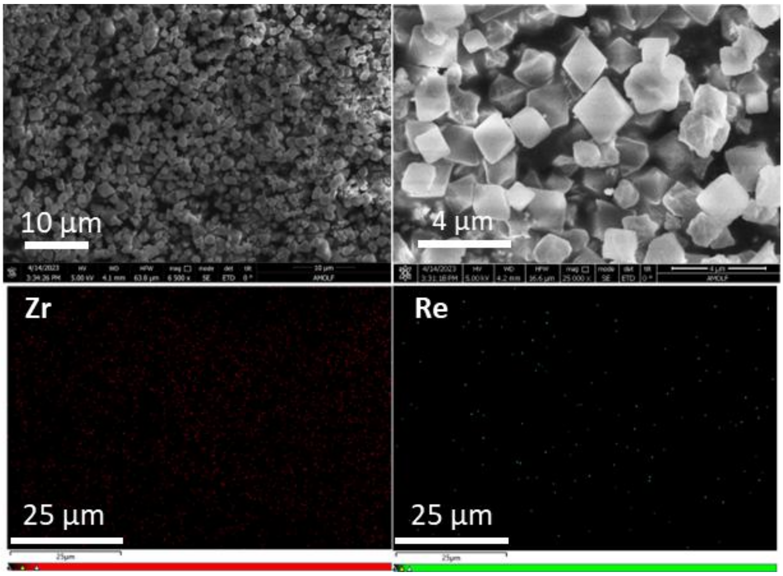


**Figure S9** SEM images (top) and EDX elemental mapping for Zr and Re (bottom) in **Re**$_{4.41}$@PCN-777

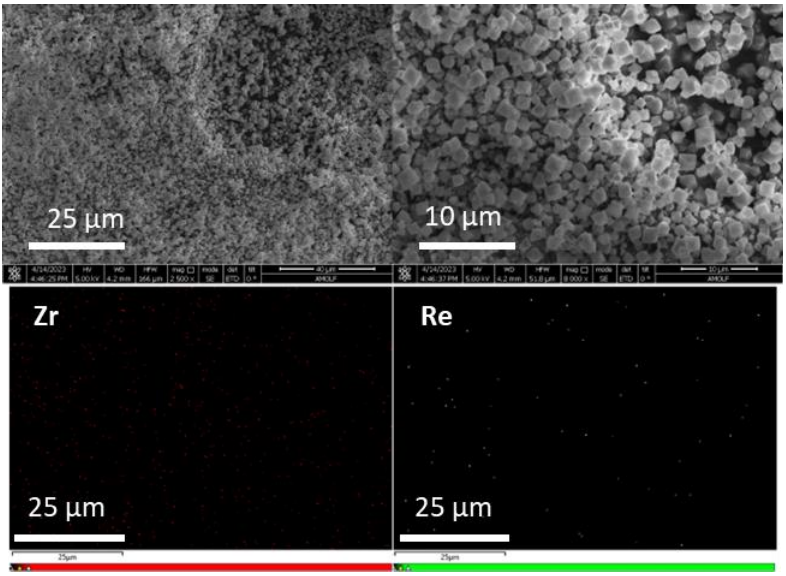


**Figure S10** SEM images (top) and EDX elemental mapping for Zr and Re (bottom) in **Re**$_{3.20}$@PCN-777

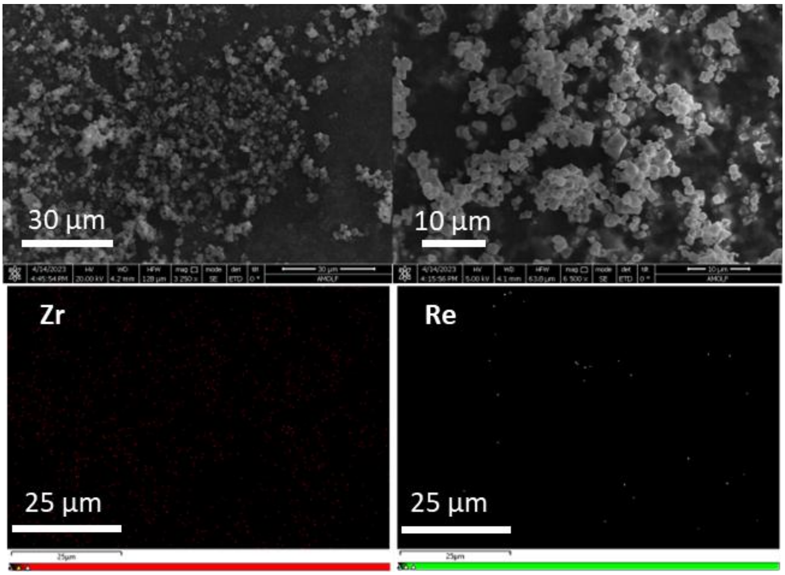


**Figure S11** SEM images (top) and EDX elemental mapping for Zr and Re (bottom) in **Re**$_{1.80}$@PCN-777

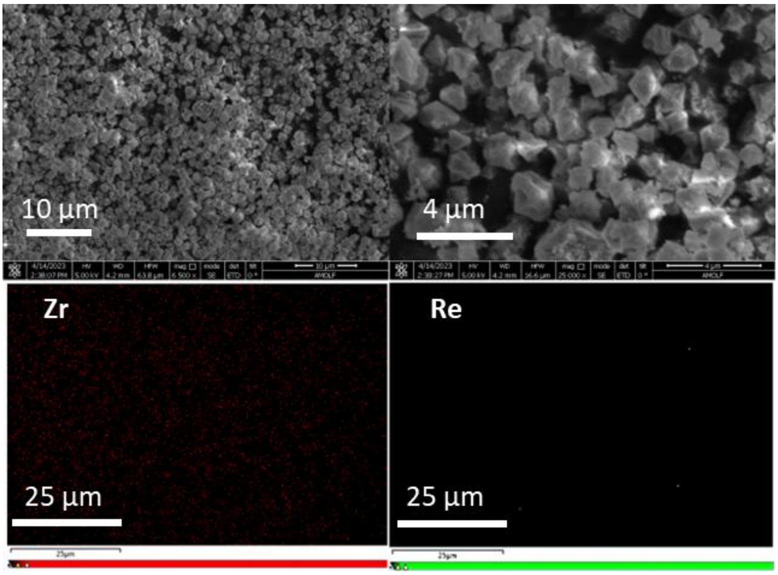


**Figure S12** SEM images (top) and EDX elemental mapping for Zr and Re (bottom) in **Re**$_{0.69}$@PCN-777

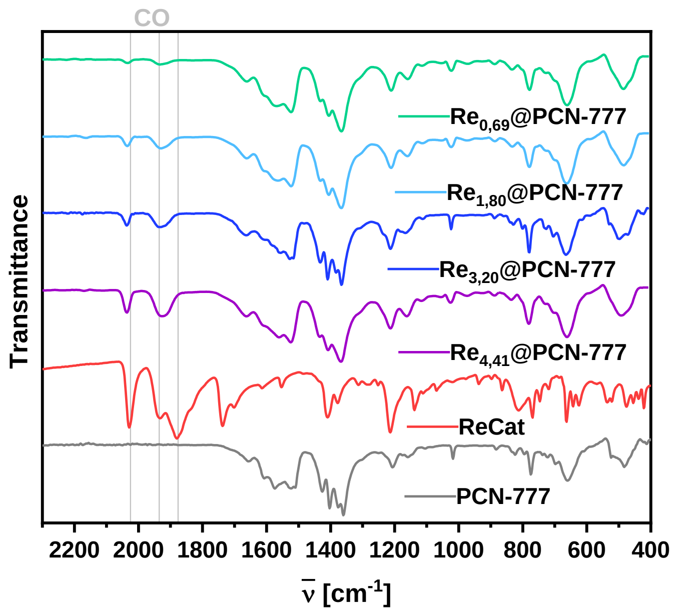


**Figure S13** ATR-FTIR spectra of pristine PCN-777, the molecular rhenium catalyst **Re** and various **Re**$_x$@PCN-777 hybrids

**Table S2** CO peaks of various **Re**-in-PCN-777 hybrids vs. the homogenous rhenium complex **Re**

| installation mode | CO peaks $\bar{\nu}$ ($cm^{-1}$) |
|---|---|
| **Re** | 2028, 1932,1880 |
| physisorption | 2023, 1916, 1893 |
| SALI | 2033, 1923 |
| electrostatics | 2046, 1940 |

Based on the data, physisorption results in the most electron-rich Re-center. Interestingly, in this case the CO bands are also shifted compared to the molecular complex, yet the signals corresponding to the vibrations of the equatorial CO ligands (1893, 1916$cm^{-1}$) are still present as two individual (partly overlapping) bands, similar as observed for **Re**, albeit with smaller half-width, which suggests a resemblance for this heterogenized catalyst with the molecular variant before immobilization. This indicates that the local environment of the MOF appears to influence the electronic properties of **Re**, despite the absence of a direct anchoring between the host and the guest, resulting in the trend depicted in Fig S13. The two A'(2) and A" vibrations fully

merge into one broad band for **Re**@PCN-777-SALI and **Re**@PCN-777-$NH_2$, which suggests a different nature of interaction between **Re** and PNC-777 compared with the physisorbed material **Re**@PCN-777.

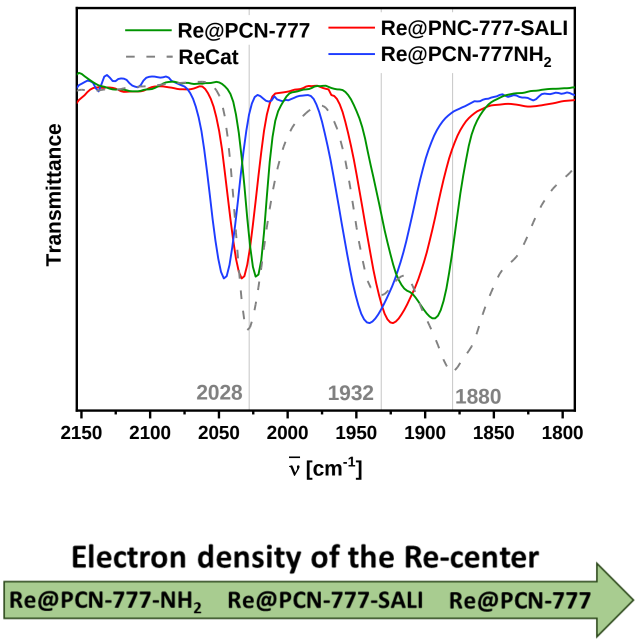


**Figure S14** Zoom-in of AIR-IR spectra for CO bands of **Re**-in-PCN-777 hybrids and **Re** (top). Schematic representation of the donating character of **Re** installation mode within PCN-777.

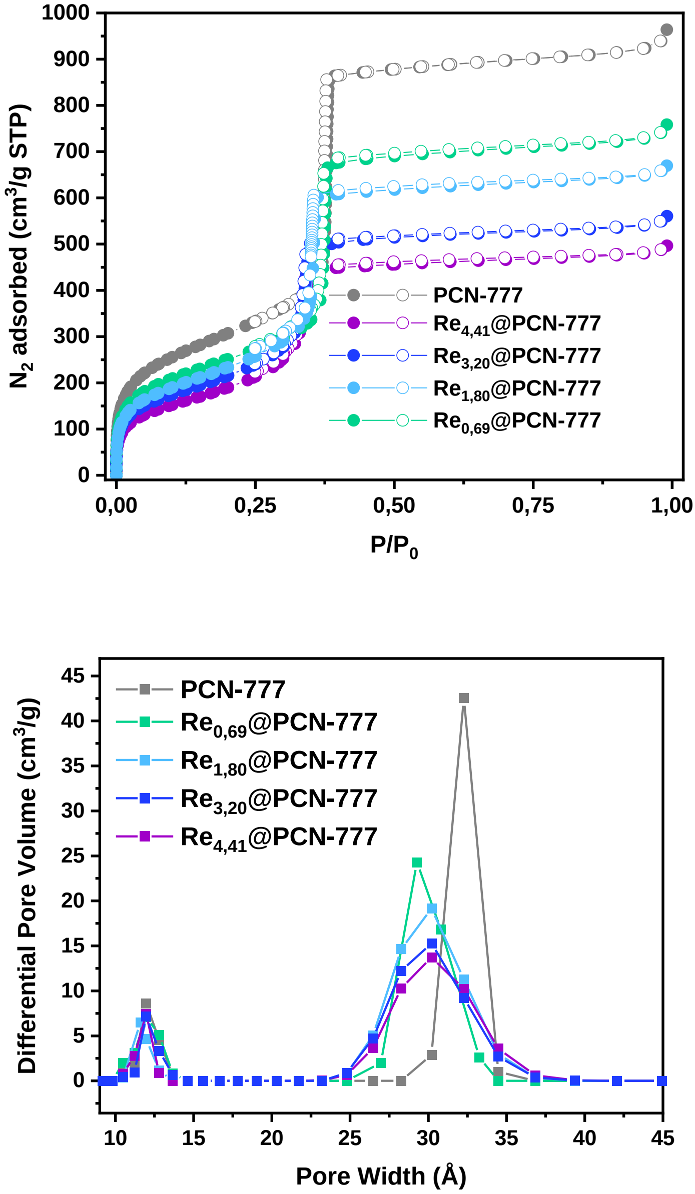


**Figure S15** Top: $N_2$ adsorption isotherms for PNC-777 and **Re**$_x$@PCN-777 hybrids (empty des. fill ads.). Bottom: Pore size distribution for PCN-777 and **Re**$_x$@PCN-777 hybrids.

## Brunauer-Emmett-Teller (BET) analysis

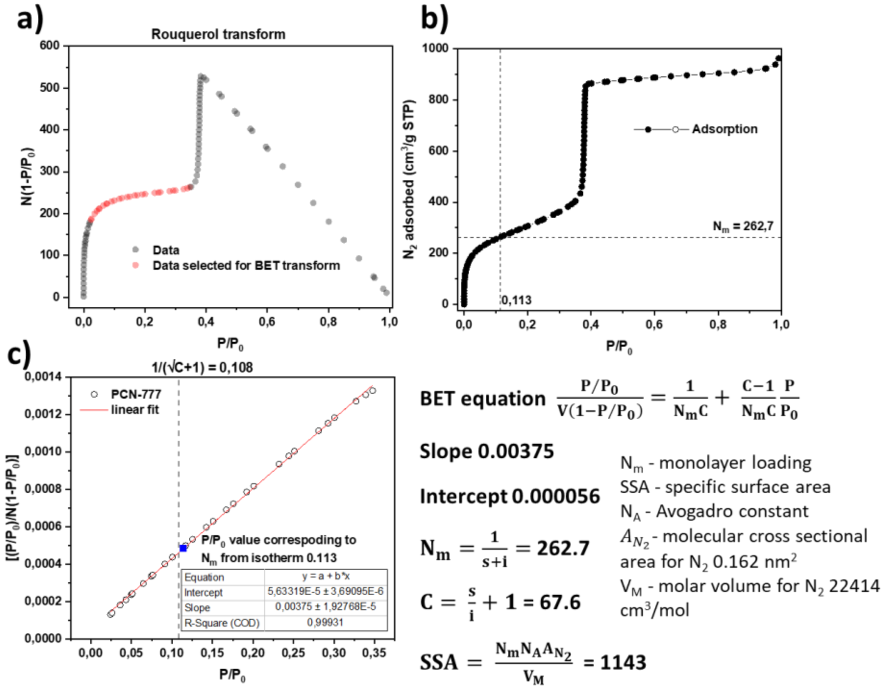


**Figure S16** Porosity analysis of the pristine PCN-777 a) Rouquerol transform plot, b) $N_2$ adsorption isotherm, c) BET transform plot

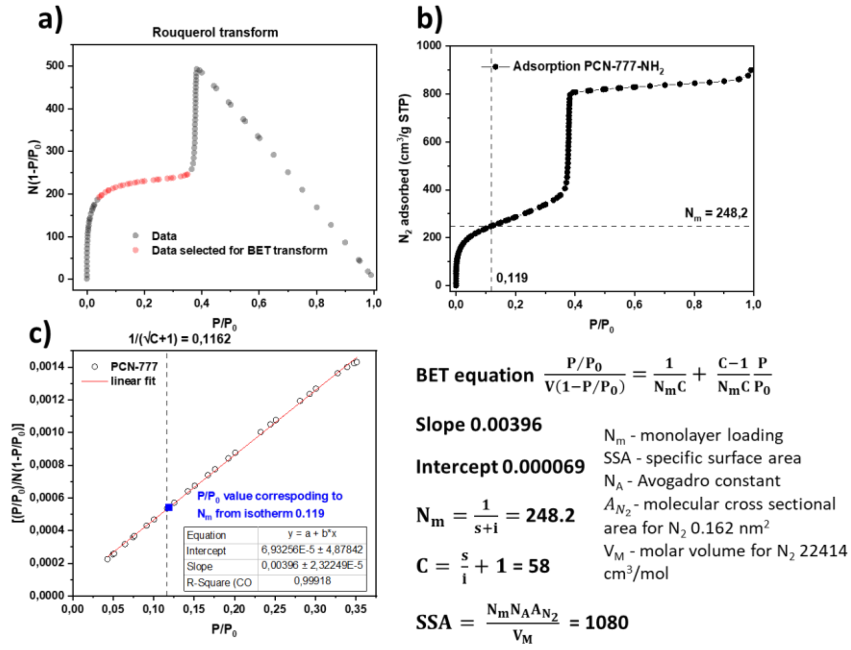


$$\text{BET equation}\quad \frac{P/P_0}{V(1-P/P_0)} = \frac{1}{N_m C} + \frac{C-1}{N_m C}\frac{P}{P_0}$$

$$N_m = \frac{1}{s+i} = 248.2$$

$$C = \frac{s}{i} + 1 = 58$$

$$SSA = \frac{N_m N_A A_{N_2}}{V_M} = 1080$$

**Figure S17** Porosity analysis of the pristine PCN-777-$NH_2$ a) Rouquerol transform plot, b) $N_2$ adsorption isotherm, c) BET transform plot

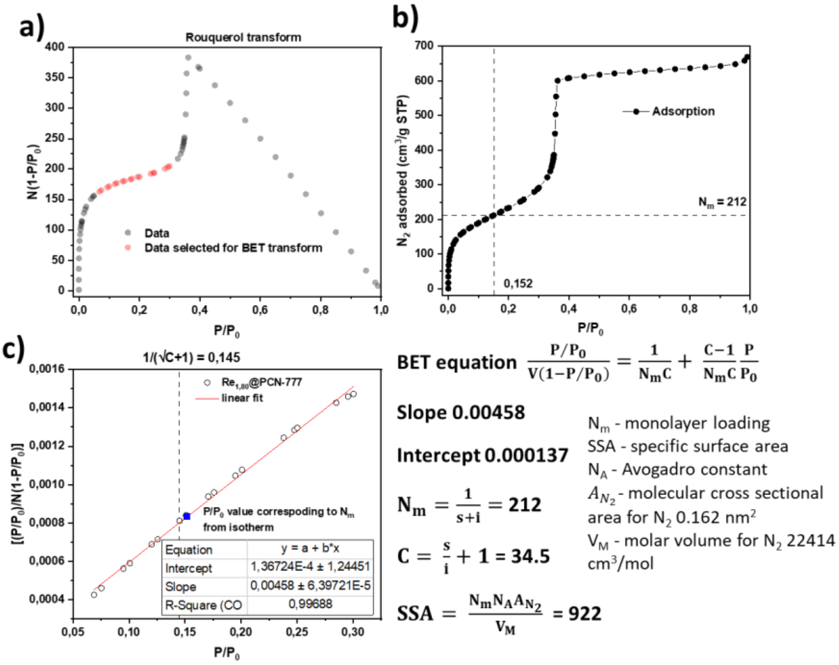


**Figure S18** Porosity analysis of **Re**@PCN-777-SALI a) Rouquerol transform plot, b) $N_2$ adsorption isotherm, c) BET transform plot

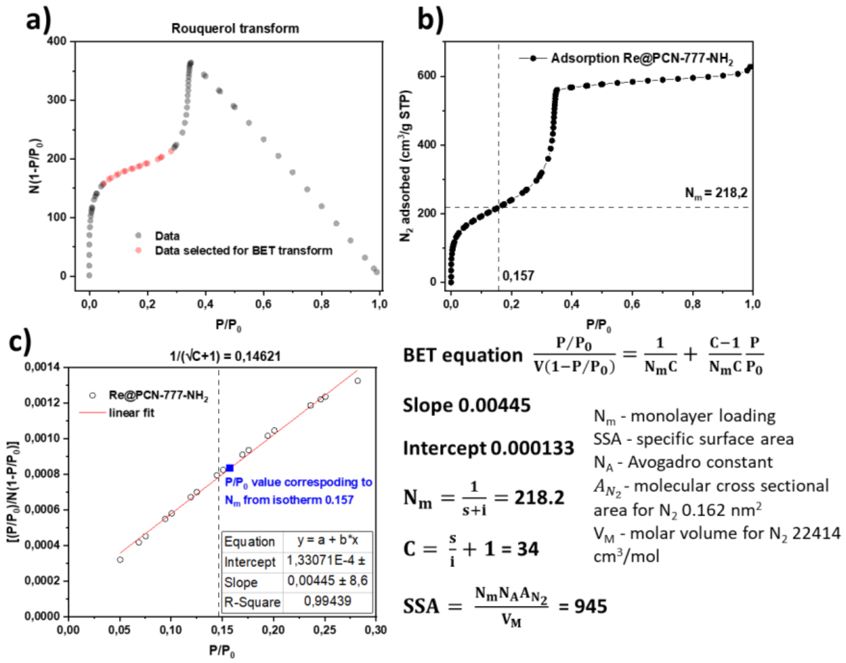


**Figure S19** Porosity analysis of **Re**@PCN-777-$NH_2$ a) Rouquerol transform plot, b) $N_2$ adsorption isotherm, c) BET transform plot

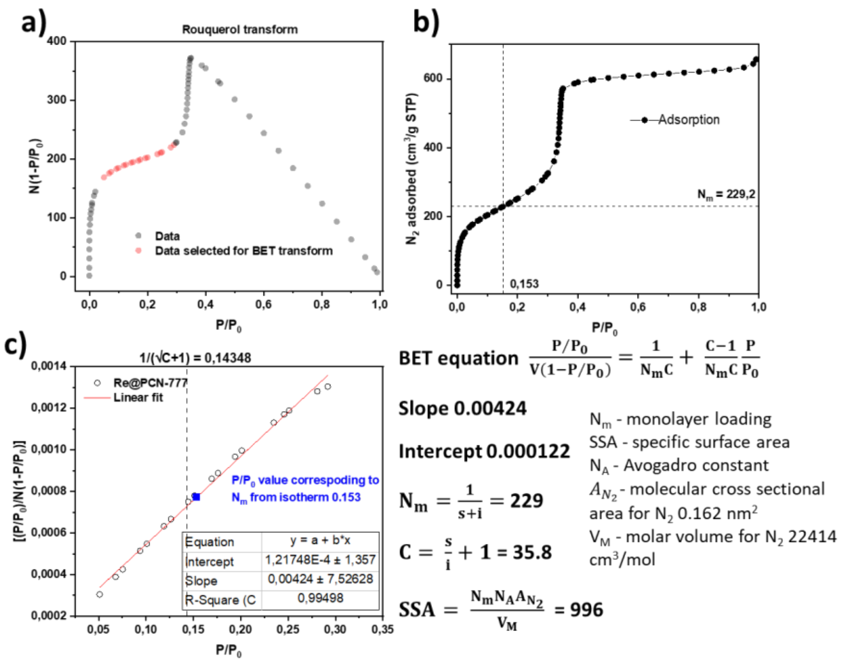


**Figure S20** Porosity analysis of **Re**@PCN-777 a) Rouquerol transform plot, b) $N_2$ adsorption isotherm, c) BET transform plot

## Brunauer-Emmett-Teller (BET) analysis for Re$_x$PCN-777

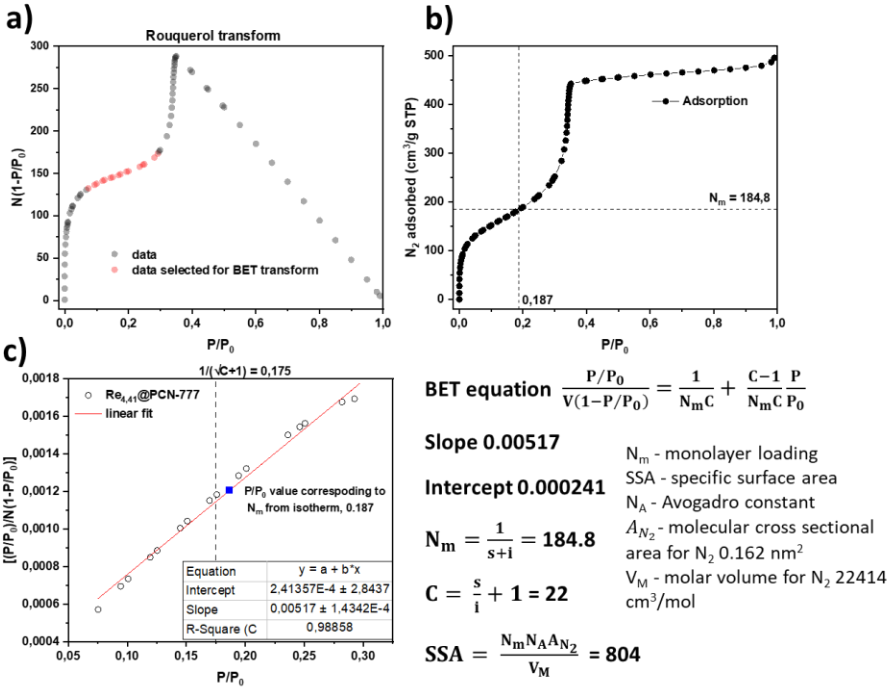


**Figure S21** Porosity analysis of **Re**$_{4.41}$@PCN-777 a) Rouquerol transform plot, b) $N_2$ adsorption isotherm, c) BET transform plot

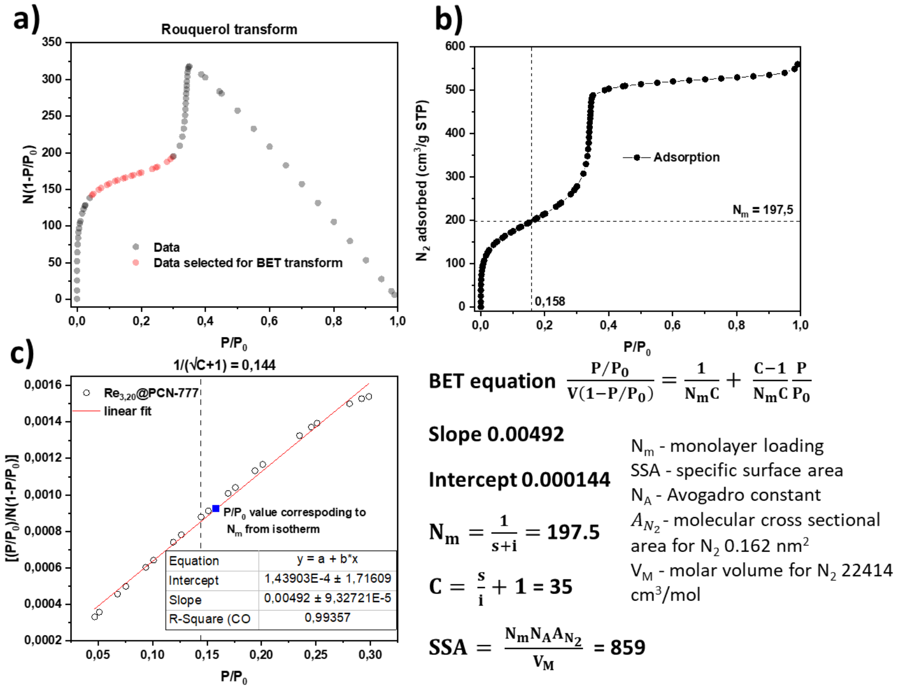


**Figure S22** Porosity analysis of **Re**$_{3.20}$@PCN-777 a) Rouquerol transform plot, b) $N_2$ adsorption isotherm, c) BET transform plot

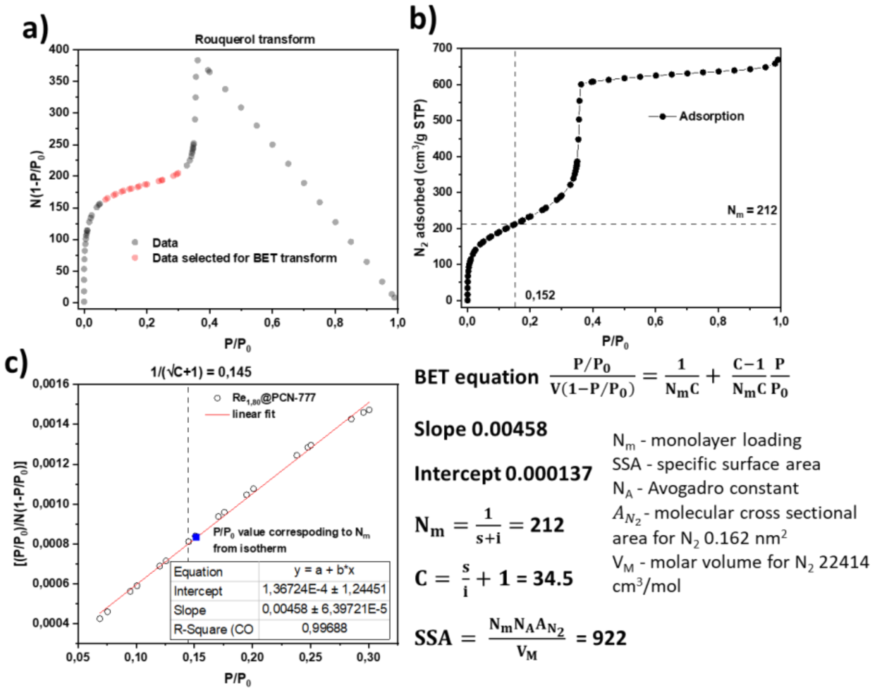


**Figure S23** Porosity analysis of **Re**$_{1.80}$@PCN-777 a) Rouquerol transform plot, b) $N_2$ adsorption isotherm, c) BET transform plot

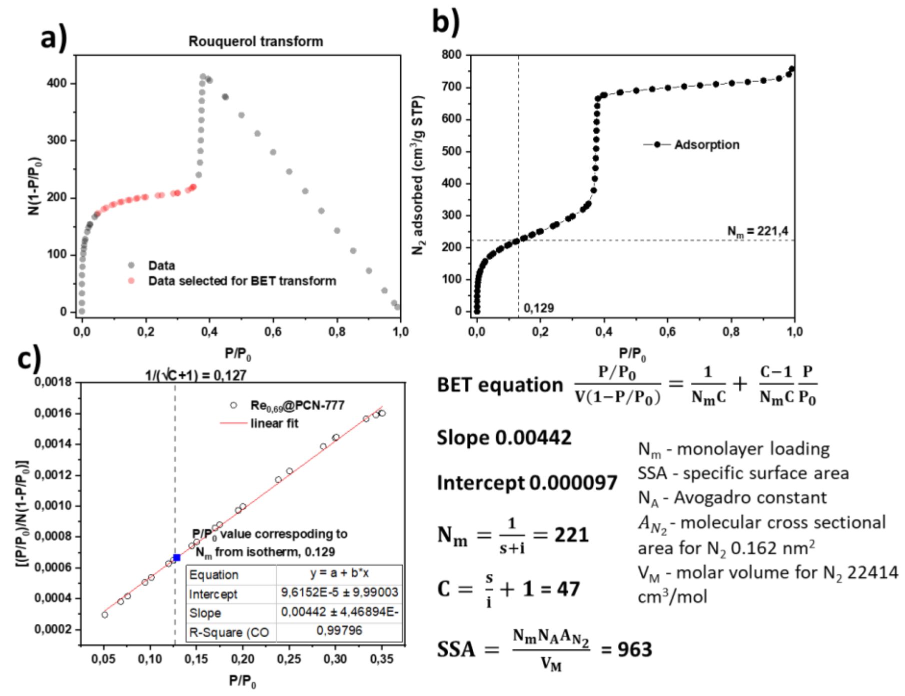


**Figure S24** Porosity analysis of **Re**$_{0.69}$@PCN-777 a) Rouquerol transform plot, b) $N_2$ adsorption isotherm, c) BET transform plot

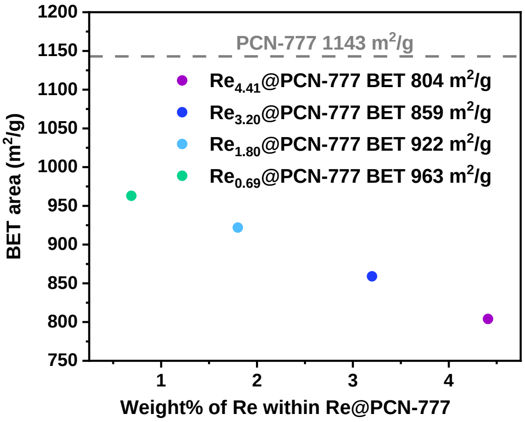


**Figure S25** BET area of **Re**$_x$@PCN-777 hybrids

**Table S3** Rhenium and zirconium content (weight percent wt%) in **Re**-in-PCN-777 hybrids before and after catalysis from ICP-MS results

| Entry | Material | Pre-catalysis [wt%] | | Post-catalysis ($2^{nd}$) [wr%] | | Post-catalysis (last) [wt%] | |
|---|---|---|---|---|---|---|---|
| | | Re | Zr | Re | Zr | Re | Zr |
| **1** | **Re**@PCN-777-SALI | 1.80 | 18.24 | 1.78 | 18.30 | 1.80 | 18.21 |
| **2** | **Re**@PCN-777-$NH_2$ | 1.79 | 19.48 | 1.41 | 19.79 | 0.95 | 20.35 |
| **3** | **Re**@PCN-777 | 1.78 | 17.42 | 0.97 | 18.22 | 0.42 | 18.76 |

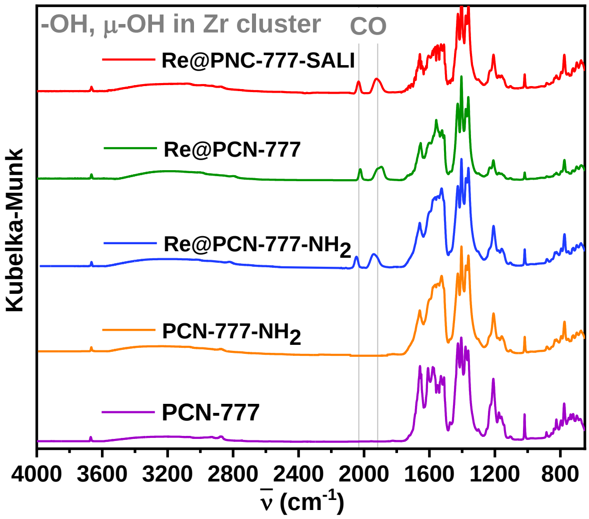


**Figure S26** Full DRIFTS spectra for PCN-777, PCN-777-$NH_2$ and **Re**-in-PCN-777 hybrids

Photocatalytic $CO_2$ reduction: Photocatalytic reactions were carried out in 30 mL air-tight pressure tubes with a small stirring bar. All reagents were placed in the reaction tube (Re@PCN-777, **RuPS**, **BIH**) and $CO_2$ saturated acetonitrile was added, then a $CO_2$ overpressure was applied reaching total 1.25 bar. The reaction tubes were placed in an in-house 3D printed "UFO reactor"[S6] equipped with a fan from the bottom and the light source from the top (Figure S1). The light source was blue Kessil LED PR160L (50 W, λ = 450 nm). The reaction headspace was analysed by GC. Each reaction was repeated at least two times and the average value was reported. (Calibration curve Figure S33).

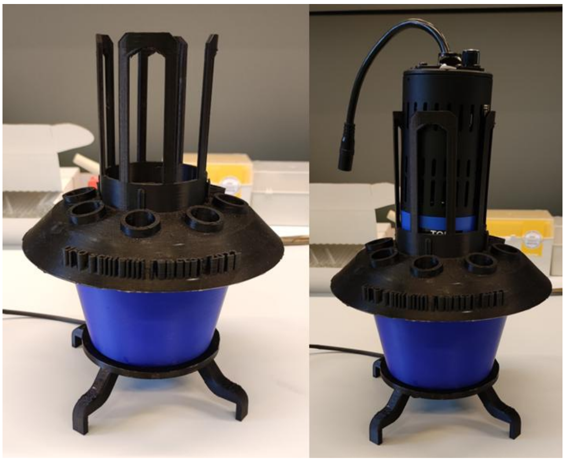

**Figure S27** „UFO reactor" for photocatalytic reactions

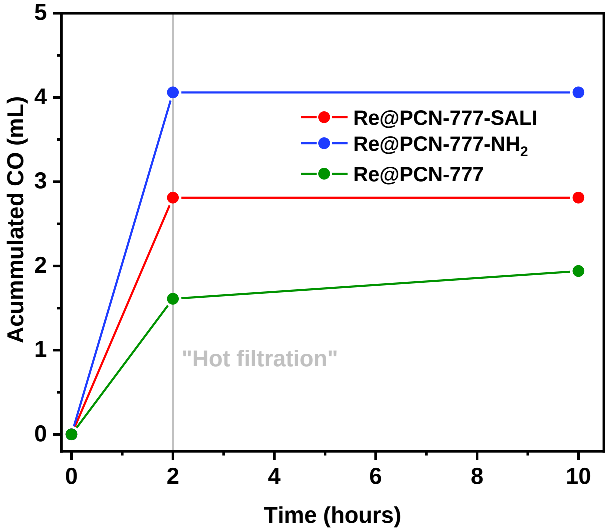


**Figure S28** "Hot filtration" test taken after two hours of irradiation of the catalytic mixture (indicated by the grey line).

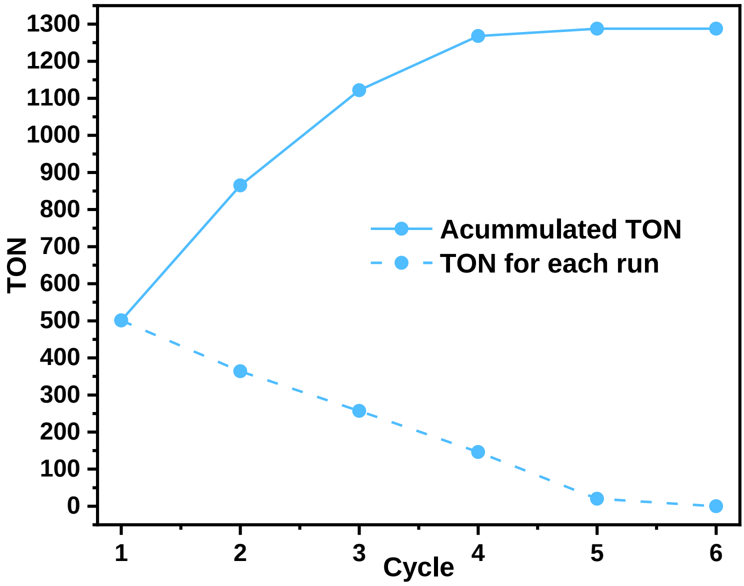


**Figure S29** TON values for each run (dashed line) and accumulated TON (solid line) obtained with $Re_{1.80}$@PCN-777 in the recyclability experiment. Conditions: 8 mL MeCN saturated with $CO_2$, $\mathbf{Re}_{1.80}$@PCN-777 (recovered),**RuPS/Re** = 5, **BIH/RuPS** = 200, 450 nm LED, 10 h for each catalytic cycle. A new portion of the RuPS was added after every cycle.

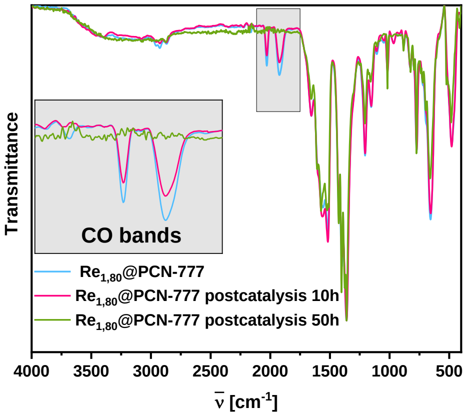


**Figure S30** ATR-FTIR spectra of pre-and post-catalysis with **Re**1.80@PCN-777

ICP-MS (after the second and the fifth run) did not reveal any changes in rhenium content for the recycled materials, compared to the pristine **Re**1.80@PCN-777 material used for the first run. In order to verify whether the effect of postulated CO dissociation in **Re**$_x$@PCN-777 (*vide supra*) is caused by light or it occurs under catalytic conditions, we carried out a control experiment wherein the hybrid **Re**1.80@PCN-777 was suspended in acetonitrile and irradiated with 450 nm Kessil LED for 10 and 50 hours. After that time, the solid was separated and probed with ATR-IR (Figure S31). The near-identical shape and intensity of the observed CO bands provide clear evidence that light irradiation itself does not lead to any chemical changes at the catalytic sites.

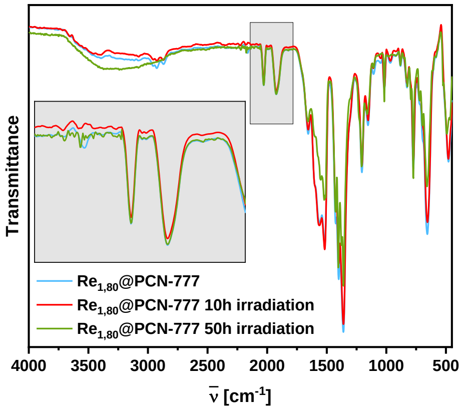


**Figure S31** ATR-FTIR spectra of **Re**1.80@PCN-777 after illumination (450 nm)

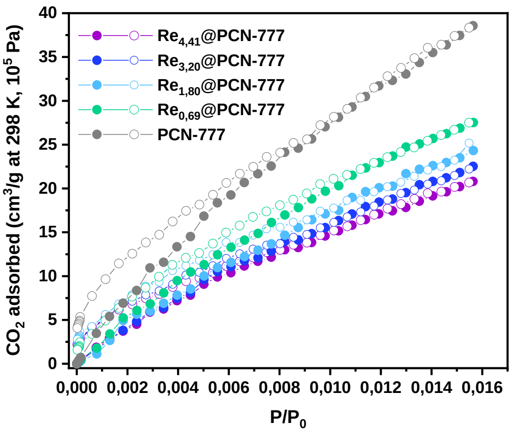


**Figure S32** $CO_2$ adsorption isotherms for PNC-777 and **Re**$_x$@PCN-777 hybrids

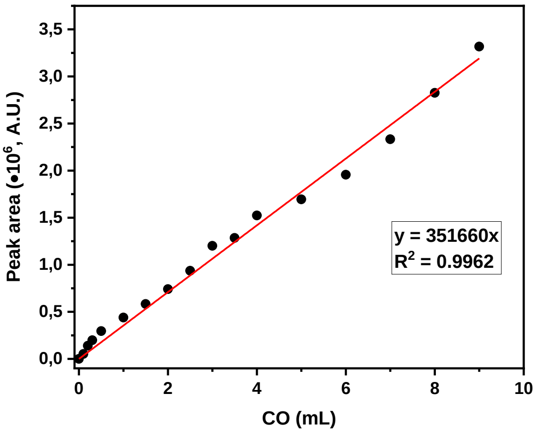


**Figure S33** GC calibration curve for photocatalysis results.

**References**

S1 D. Feng, K. Wang, J. Su, T-F. Liu, J. Park, Z. Wei, M. Bosch, A. Yakovenko, X. Zou, H-C. Zhou, *Angew. Chem. Int. Ed*. **2015**, *54*, 149-154.

S2 a) P. Llewellyn, G. Maurin, J. Rouquerol, in *Adsorption by Powders and Porous Solids*, 2nd ed.; F. Rouquerol, J. Rouquerol, K. S. W. Sing, P. Llewellyn, G Maurin, Eds.; Academic Press: Oxford, **2014**; p 565; b) D. A. Gómez-Gualdrón, P. Z. Moghadam, J. T. Hupp, O. K. Farha, R. Q. Snurr, *J. Am. Chem. Soc*. **2016**, *138*, 215−224.

S3 a) J. M. Smieja, C. P. Kubiak, *Inorg. Chem.* **2010**, *49*, 9283-9289; b) J. Hawecker, J.-M. Lehn, R. Ziessel, *J. Chem. Soc. Chem. Commun*. **1983**, *9*, 539-538.

S4 a) E. Mühlbauer, A. Klinkebiel, O. Beyer, F. Auras, S. Wuttke, U. Lüning, T. Bein, *Microporous Mesoporous Mat*. **2015**, *216*, 51-55; b) M. Köppen, O. Beyer, S. Wuttke, U. Lüning, N. Stock, *Dalton Trans.* **2017**, *46*, 8658-8663.

S5 Z. Lu, J. Liu, X. Zhang, Y. Liao, R. Wang, K. Zhang, J. Lyu, O. K. Farha, J. T. Hupp, *J. Am. Chem. Soc*. **2020**, *142*, 21110-21121.

S6 T. M. Masson, S. D. A. Zondag, J. H. A. Schuurmans, T. Noël, *React. Chem. Eng.* **2024**, *9*, 2218-2225.
